# 3D-printed sustainable biocomposites via valorization of biomass: focus on challenges and their future perspectives

**DOI:** 10.1007/s11356-025-37109-5

**Published:** 2025-11-01

**Authors:** Ashish Soni, Sonu Kumar Gupta, Dhinakaran Veeman, Jitendra Kumar Katiyar

**Affiliations:** 1 Centre for Additive Manufacturing, Chennai Institute of Technology, Chennai, Tamil Nadu 600069 India; 2 Department of Civil Engineering, Institute of Engineering and Technology, Sandip University, Nashik, MH 422212 India; 3 Department of Mechanical and Industrial Engineering, Manipal Institute of Technology, Manipal Academy of Higher Education, Manipal, 576104 Karnataka India

**Keywords:** Biocomposites, Biomass, Circular economy, Degradation, 3D printing, Sustainability

## Abstract

Ineffective management of plastic wastes and biomass is a global concern. The annual generation of plastic waste is around 380 million tonnes, with only 9% being recycled, and the production of biomass is 140 billion metric tonnes. The worry of the environment and demand for sustainability has triggered the configuration of biocomposites as an alternative to conventional materials. 3D printing is a promising technique for the manufacturing of polymer-based composites. The review has integrated 3D printing, utilization of biopolymers, and biomass. The work critically reviewed the various aspects of biocomposites. The characteristics of biocomposites, including processing, fabrication techniques, 3D printing parameters, environmental degradation, and applications, are discussed. The allied works on biocomposites are summarized. The challenges and opportunities of biocomposites are identified. In essence, the review encapsulates the transformative potential of 3D printing with biodegradable plastics and biomass for advancements of biocomposites and accentuates its potential in fostering sustainability. The review has revealed that 3D-printed biocomposites are potent for diverse engineering applications. The biocomposites are economical and eco-friendly and reduce energy consumption and emissions of greenhouse gases. The biomass-based 3D-printed biocomposites have shown comparable mechanical properties to traditional materials. The review revealed that different printing parameters substantially influence the strength, flexibility, crystallinity, and dimensional accuracy of printed parts. The optimization of 3D printing parameters is crucial for the improvement of performance. Biocomposites can overcome the challenges of the linear economy by transforming the wastes into valuable resources.

## Introduction

**Fig. 1:**
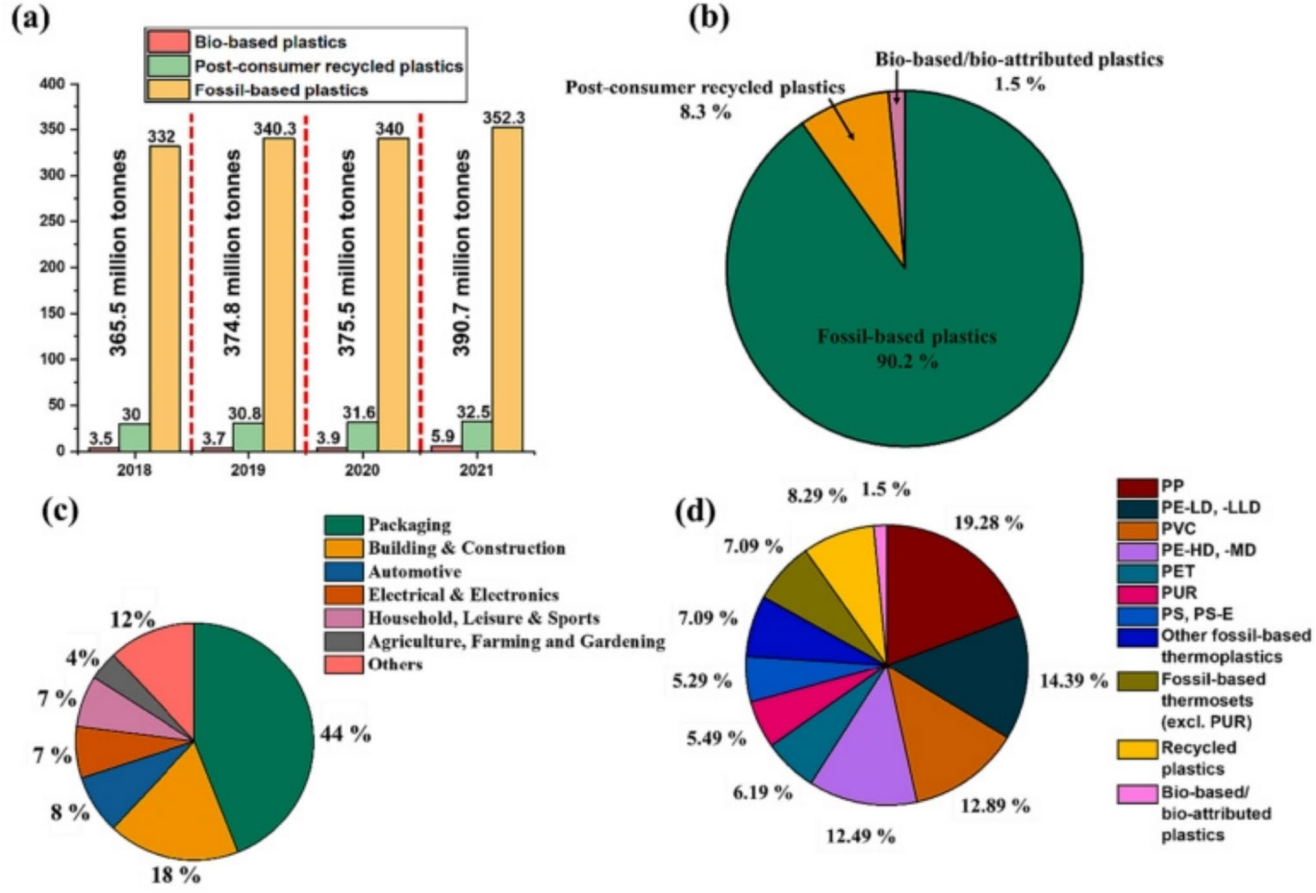
**a** Global plastic production trends from 2018 to 2021. **b** Composition of global plastics production in 2021. **c** Breakdown of global plastics use by application in 2021. **d** Plastic-type breakdown in 2021 (Hassan et al. 2023)

Globally, the rate of generation of solid waste is increasing exponentially. Solid waste is composed of a variety of materials containing plastics, paper wastes, construction and demolition wastes, food waste, agro wastes, etc. Among the generated solid waste, plastics are the cause of concern due to their wide generation, low biodegradability, and inefficient recycling practices which are deteriorating the environmental condition perpetually (Kumar et al. 2021). The manufacturing of plastics has increased rapidly, and the global production of plastic wastes is anticipated to be 260–460 metric tonnes annually (Borrelle et al. 2020). Moreover, the amount of plastic generated by 2050 will surpass 500 million metric tonnes, which is more than 33 times what was produced in 1960 (Wang et al. 2023). The worldwide manufacturing of plastic between the period 2018 and 2021, the different types of plastics, along with the distribution and application of plastic in 2021, are shown in Fig. 1a–d, which reveals that about 90.2% of the plastics produced globally in 2021 is derived from fossil fuels. Conversely, biobased and recycled plastic products occupy a small fraction of about 1.5% and 8.3%, respectively (Hachem et al. 2023). Despite the tremendous effort to eliminate plastic wastes, the generation of plastics has not been arrested due to their remarkable characteristics and diverse applications. It is reported that merely 9% of the total plastic waste is processed, whereas incineration constitutes 12% of the total plastic waste, and the remaining 79% of the plastic waste is answerably dumped in landfills or water bodies, which negatively influences the lives of humans as well as marine aquatics (Luhar and Luhar 2019). Biodegradable polymers, also known as eco-friendly or biobased polymers, are a promising solution to plastic wastes (Dananjaya et al. 2022a). These polymers can degrade naturally in the environment and can be a substitute for conventionally produced plastics (Keskin et al. 2017). The different biopolymers reveal a wide domain of characteristics such as respectable mechanical properties, thermal properties, and barrier characteristics (Post et al. 2021).

**Fig. 2:**
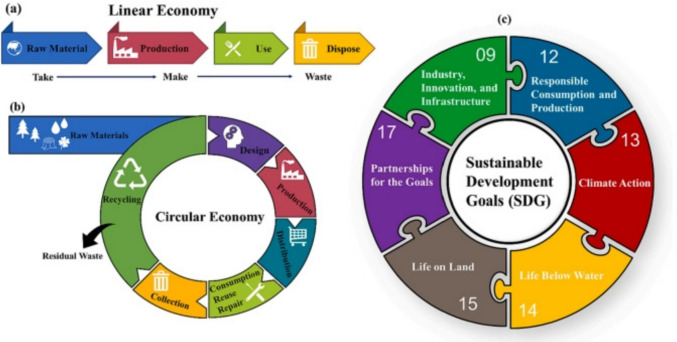
The sequential steps involved in **a** linear economy, **b** circular economy, and **c** Sustainable Development Goals through 3D-printed biocomposites (Hassan et al. 2023)

Apart from plastic wastes, due to the huge generation of organic waste, biomass is also a cause of worriment to the environment. Agricultural and forestry are the major producers of biomass, with farming only constituting about 140 billion MT of biomass annually (Lim et al. 2022). Metropolitan solid waste is a notable origin of biomass, with an annual production of roughly 2.01 billion tons of biomass (Kibria et al. 2023). The ineffective treatment of the generated biomass is challenging the environment. The implementation of the circular economy (CE) by reprocessing, reutilizing, and retrieving resources could offer a promising solution to the generated waste (Yang et al. 2022). Figure 2a and b represents the consecutive steps associated with the liner and circular economy, respectively. The utilization of biomass and biopolymers in biocomposites promotes sustainability in modern industries.

A considerable advancement has been noticed for biocomposites for the treatment, characterization, specific utilization, and employment of different unused materials. The biomass-derived reinforcements can effectively replace the synthetic reinforcement carbon fiber, glass fiber, metallic and inorganic fillers, etc. (Bhatia et al. 2019). The characteristics of the bioreinforcements are influenced by the method of extraction. Biocomposites have a huge potential to improve the living conditions in rural communities and create jobs through the collection, processing, and transportation of new materials (Satyanarayana et al. 2009). Several studies have been conducted on the structural and operational properties of fibers reinforced with various thermoplastics such as poly (hydroxy alkanoates) (PHA) and polylactic acid (PLA) as well as thermoset substances such as polyester and epoxy bonded with fibers (Yu et al. 2014; Forintos and Czigany 2019; Fraternali et al. 2011). Biocomposites are subjected to different applications in the locomotive, wrapper, electronics, aerospace, and civil sectors (Zini and Scandola 2011). Biocomposites have several distinguishing characteristics over traditional materials including fairly particular mechanical characteristics, heat insulation, CO_2_ independence, excellent damping characteristics, significant fatigue and wellness security accessibility, excessive abrasive and corrosion-resistant properties, minimal density, little production energy, and lightweight (Andrew and Dhakal 2022). The applications of biocomposites have grown significantly in recent years. Moreover, the utilization of uncommon biomaterials can open up a whole new-fangled value sequence for the yields. In actuality, biocomposite can provide immediate socio-economic benefits in countryside communities, especially in advanced regions where these resources are plentiful.

The different biocomposites have limitations of low damp resisting capacity, incompatibility towards fibers and matrix, logistical glitches with the request, less fire resistance, trouble in handling, and highly anisotropic characteristics, i.e., fiber variability. Furthermore, the characteristics of a biofiber depend upon the derived sources, which affect the durability and other attributes of biocomposites (Stamboulis et al. 2001). To achieve long-term durability and characteristics, the issues associated with materials and production essentially need to be addressed. Biofibers encompass huge quantities of cellulose, hemicelluloses, pectin, lignin, and additional hydrophilic constituents which are responsible for poor interfacial bonding with hydrophobic polymer (Jawaid and Khalil 2011). These properties can lead the biocomposite to lose its mechanical and thermal rigidity, limiting its usage in applications that carry loads throughout a wide range of industrial applications. Numerous strategies have been used, including surface modification, fabrication techniques, hybridization strategies, and nano-engineering, to address the issue of compatibility in biocomposites (Huda et al. 2008).

3D printing presents a great chance to put the concepts of the circular economy into action by lowering the need for virgin resources, allowing the use of reused materials, expediting the creation and manufacturing process, and boosting local and decentralized manufacturing (Rose and Bharadwaj 2023). This approach has a revolutionary effect on the transformation of waste plastic and biomass, which coincides with several Sustainable Development Goals (SDGs), as shown in Fig. 2 (c). The different thermoplastic materials, including nylon, thermoplastic urethane (TPU), polylactic acid (PLA), acrylonitrile butadiene styrene (ABS), polystyrene (PS), polypropylene (PP), polyethylene (PE), polyethylene terephthalate (PET), polycarbonate (PC), polycaprolactone (PCL), polyether ether ketone (PEEK), and polymeric acid (PMA), can be used to fabricate a 3D-printed product. Due to its capacity to create intricate shapes and geometries (Bi and Huang 2022), less waste of material (Nadagouda et al. 2020), less energy consumption (Shuaib et al. 2021), and enable on-demand production, this technology has been incredibly popular in recent years (Mahshid et al. 2023). By 2026, it is projected that the worldwide 3D printing market will have grown to 23.33 USD billion (Hassan et al. 2023). There are numerous benefits to the environment from 3D printing with recycled plastic, including the possibility of lower waste and energy usage. When plastic is recycled instead of being processed and manufactured as virgin plastic, carbon emissions can be reduced by 30% to 80% (Schwarz et al. 2021). Direct Ink Writing (DIW) is an extrusion-based AM technique that deposits the materials in semi-liquid form or viscous layers to generate a complex 3D-printed structure. The DIW technique can effectively print different types of materials, namely, ceramics, metals, and polymers, by modifying the rheology of the ink. The ink is allowed to dispense from a nozzle into a substrate. The solidification of the materials takes place through evaporation and photopolymerization. The DIW consists of three components, including a computer-aided system, an extrusion needle, and an X–Y–Z platform. CAD designs the structure and converts it into a code language. The extrusion needle ejects the ink under suitable pressure from nitrogen, and the X–Y–Z platform moves under computer instruction to run the extrusion system (Li, Li, & Li, 2015). The researchers have developed 3D-printed sodium-ion batteries with the advancement of advanced electrode (He et al. 2024). Moreover, the 3D-printed solid-state batteries using a ferroelectric BiFeO3 (BFO) filler for the poly(ethylene oxide) (PEO)-based electrolyte were introduced (Han et al. 2025).

Table 1 presents a comparison of the most often utilized procedures, together with the details on their description, cost, material requirements, benefits, and drawbacks, to aid in a comprehensive understanding of the different approaches.

**Table 1:** Comparison of different 3D printing techniques

S. No.	3D printing techniques	Details	Materials used	Cost	Advantages	Limitations
1	Fused deposition modeling (FDM)	Melting of filament through extruder to build objects layer by layer (Shaqour et al. 2021)	Thermoplastics, metals, and composites	Low	Easy to use (Zhao et al. 2019c), Print a wide range of materials (Srinivasan et al. 2020)	Limited resolution and surface finish (Mathew et al. 2023), Layer adhesion can be weak (Garzon-Hernandez et al. 2020)
2	Stereolithography (SLA)	Liquid resin cured using a light source or laser layer by layer (Weng et al. 2016)	Photopolymer resin	High	High resolution (Layani et al. 2018), Smooth surface finish (Manoharan et al. 2013)	Limited build size (Ho et al. 2015), Expensive equipment and materials (Gu et al. 2016)
3	Selective Laser Sintering (SLS)	Selectively fuse powdered material by using a laser layer by layer (Yuan et al. 2018)	Nylon, Thermoplastic polyurethane (TPU), and other plastics, ceramics, and metals	Moderate to high	Wide range of materials (Gibson and Shi 1997), Complex geometries (Malashin et al. 2024)	Rough surface finish, Requires post-processing (Bacchewar et al. 2007)
4	Directed Energy Deposition (DED)	Uses a focused energy source to melting and fuse a material feedstock	Primarily uses metals in powder or wire form but it can also deposit polymers and ceramics	High	High build rates and mechanical strength suitability for large parts, multi-material capability, and reduced material waste (Dezaki et al., 2022)	Low build resolution, requiring extensive post-processing like machining to achieve smooth surfaces (Jandyal et al., 2022)
5	Direct Ink Writing (DIW)	AM technique that builds 3D structures by extruding a paste-like, liquid or semi-solid "ink" through a nozzle onto a surface, depositing it layer-by-layer	Wide range of materials including ceramics, metals, polymers, and composites	Low	Versatility in materials, high precision and resolution, enabling the fabrication of intricate details (Pak et al., 2025)	Slow Speed & Scalability, Size Constraints, Requires Post-Processing (Sevcik et al., 2024 )
6	Powder Bed Fusion (PBF)	AM process to create objects layer by layer by selectively fusing powdered material using a heat source, such as a laser or electron beam	Metals, polymers and even ceramics and pharmaceutical materials	High-cost	Reduces material waste through powder recycling, High resolution and reducing tooling costs (Awad et al., 2021)	High equipment and material costs, Requires extensive post-processing, limited build volume and build speeds for large-scale production (Ding et al., 2023 )

The literature has addressed reprocessing methods and 3D printing. However, there is still a dearth of thorough assessments on the incorporation of 3D printing and the use of biomass as reinforcement with biopolymer in biocomposites for structural purposes. This research intends to fulfill this gap by investigating the potential of 3D printing technology in utilizing biomass for biocomposites in which biopolymers act as a matrix and waste biomass serves as reinforcement. This article provides a critical overview of recent research on several areas of biocomposites such as characterization, environmental attack, applications, and prospects. The various processes for the development of biocomposites are presented. Moreover, the factors affecting the development of biocomposites, economic viability, and environmental impact of biocomposites are outlined. The effects of 3D printing parameters, associated challenges, and upcoming predictions of biocomposites are discussed. The extensive literature review will provide several valuable insights into 3D printing and upcycling of biomass with biopolymers besides discussing the goals, prospects, and possible paybacks of the expertise for sustainability. The examination will benefit researchers and authorities who make decisions, producers, and scientists working in the field of sustainability. The evaluations of the work will help to enhance environmentally friendly waste disposal methods, as well as the use of 3D printing technology to generate biocomposites from biomass and recyclable plastics. This review will serve as an essential foundation for further investigation and industrialization of biocomposites.

## Biodegradable polymer

**Fig. 3:**
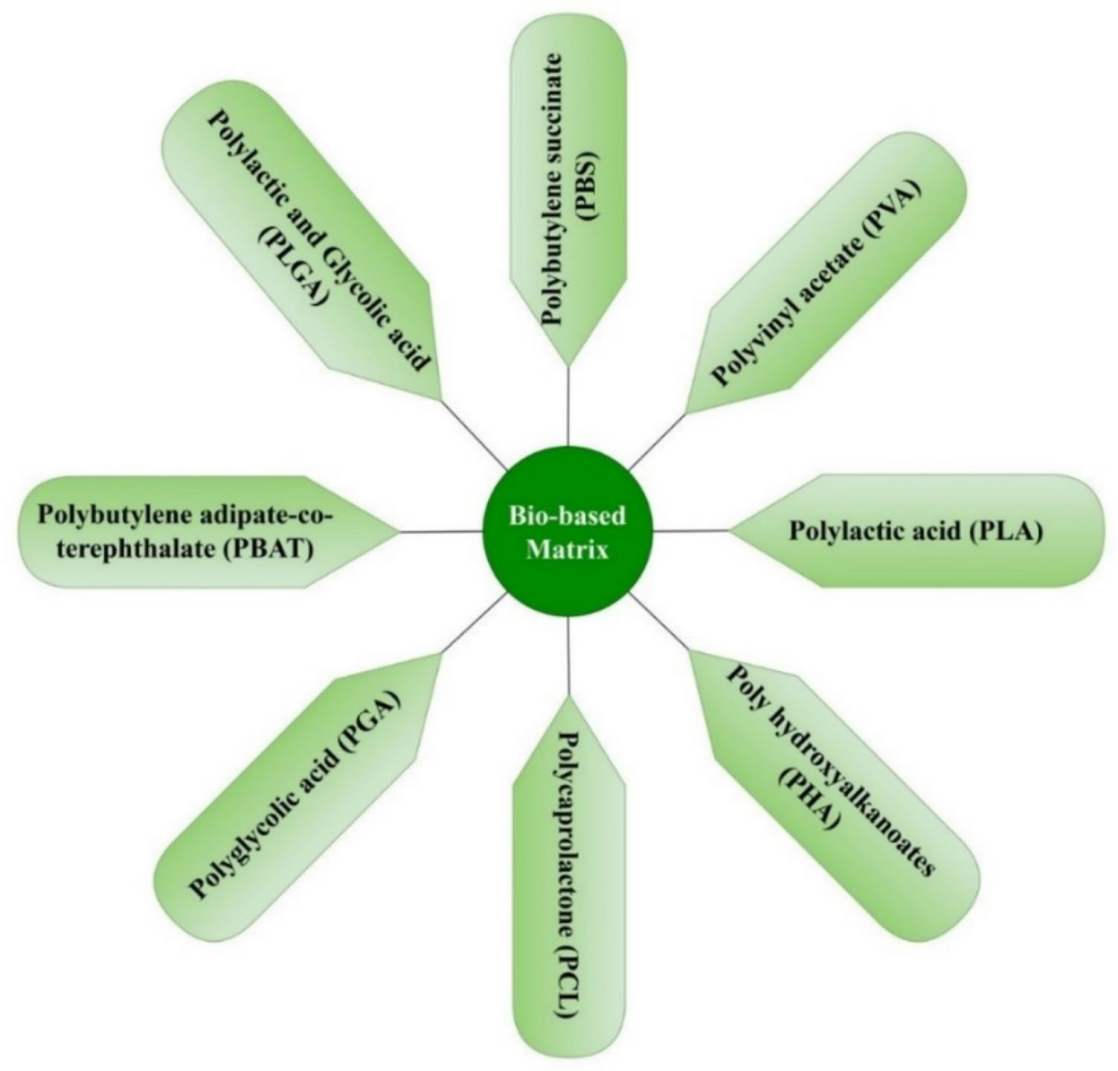
Different types of biopolymers

Biodegradable polymers are also known as biopolymers or eco-friendly polymers, which can be efficient alternatives to mitigate the plastic leftover catastrophe, and these polymers generally degrade into the environment with time (Dananjaya et al. 2022b). These types of polymers can be sustainable alternatives instead of petroleum-based traditional plastics (Keskin et al. 2017). In this regard, various biobased polymer matrices, such as polyhydroxyalkanoates (PHA), polylactic acid (PLA), polycaprolactone (PCL), polybutylene adipate-co-terephthalate (PBAT), and starch-based polymers, etc., as shown in Fig.3, are available to facilitate means of promising alternatives (Zhuge et al. 2022). These biobased polymers impart a range of mechanical properties and thermal properties. Biobased polymers have various applications, like packing, farming, biomedical applications, and buying goods, which facilitates diminishing greenhouse effects and promoting sustainability (Post et al. 2021). The environment-friendly materials may have several benefits in favor of environmental protection, and related work leads to futuristic advancement in the same. The properties of different biopolymers, monomers, and processing are listed in Table 2.

**Table 2:** Properties and processing of biopolymers and their monomers

S. No.	Types of biobased polymers	Monomers/abbreviations	Production techniques		Source of polymers	Extrusion temp. (°C)	Properties	Sources
1	Polyhydroxalkanoates (PHA)	ω-Hydroxalkanoates/polyhydroxalkanoates		Bacterial fermentation	Sugars with biosynthesis	160	High stiffness, UV stability	(Mehrpouya et al. 2021)
2	Polylactic acid (PLA)	Lactic acid/polylactic acid		Condensation polymerization, lactic ring opening	Plant starch	160–222	High strength and toughness	(Kovalcik 2021)
3	Polyvinyl acetate (PVA)	Styrene/poly vinyl acetate		Free radical polymerization	Petroleum	190–210	Excellent barrier properties, water solubility	(Basa et al. 2021)
4	Polycaprolactone (PCL)	ε-Caprolactone/polycaprolactone		Ring opening polymerization	Petroleum and starch	Up to 120	High strength, biocompatibility	(Popescu et al. 2023)
5	High impact polystyrene (HIPS)	Styrene/high impact polystyrene		Suspension polymerization	Petroleum	230–245	High heat resistance, durability, and impact resistance	(Sajjadi et al. 2022)
6	Polyglycolic acid (PGA)	Glycolic acid/polyglycolic acid		Condensation and ring opening	Plant sugars	220	Excellent gas barrier, high strength	(Kundak and Bilisik 2023)
7	Polylactic glycolic acid (PLGA)	Lactic and glycolic acid/polylactic and glycolic acid		Ring-opening copolymerization	Aliphatic polyester	110–140	High strength, biocompatibility	(Sun et al. 2022)
8	Polybutylene succinate (PBS)	Succinic acid and1,4-butanediol/polybutylene succinate		Polycondensation	Starch	160–200	Strong barrier properties	(Dönitz et al. 2023)
9	Polybutylene adipate-co-terephthalate (PBAT)	1,4-Butanediol and adipic acid and the polymer of DMT with 1,4-butanediol/polybutylene adipate-co-terephthalate		Condensation polymerization	Starch	140–170	Biodegradability, flexibility	(Ulbrich et al. 2022)

### Sources of biodegradable polymers

**Fig. 4:**
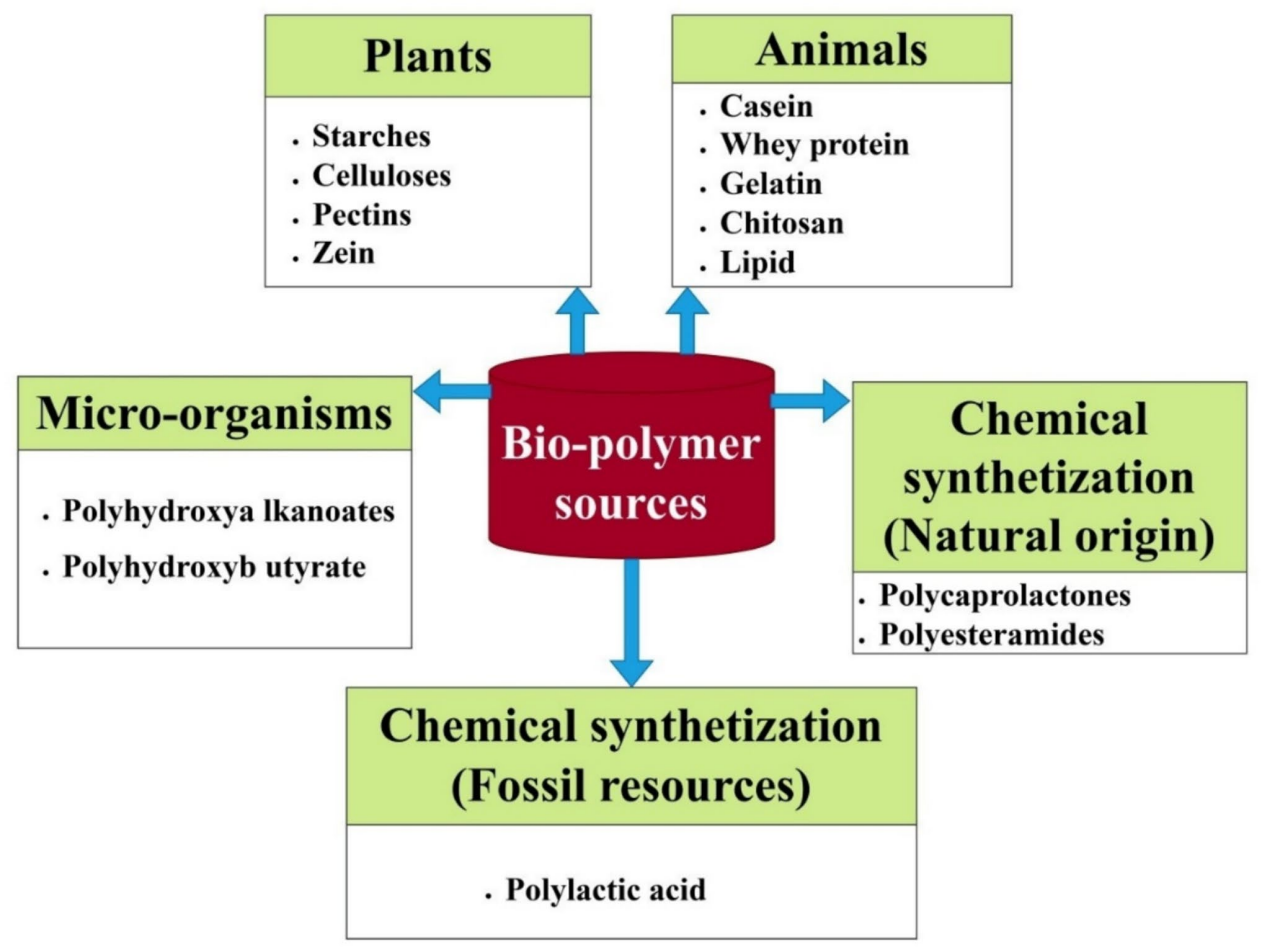
Sources of biopolymers

Biodegradable polymers are developed by using material from agriculture (plants), microbes (fungi), and bioderived monomers (Vinayagamoorthy and Venkatakoteswararao 2020). Significant polysaccharides include cellulose, chitin, alginate, xanthan gum, dextrin, and carrageenan (Luzi et al. 2019). Starch is seen as a viable substance since it has attractive qualities of availability, thermoplastic behavior, recycling potential, and sustainability (Nevoralová et al. 2019). The sources of biodegradable polymers can be divided into five different ways, which are presented in Fig. 4. These polymers can be obtained from plants, animals, micro-organisms, chemical synthetization (natural origin), and chemical synthetization (fossil resources).

### Classification of biodegradable polymers

**Fig. 5:**
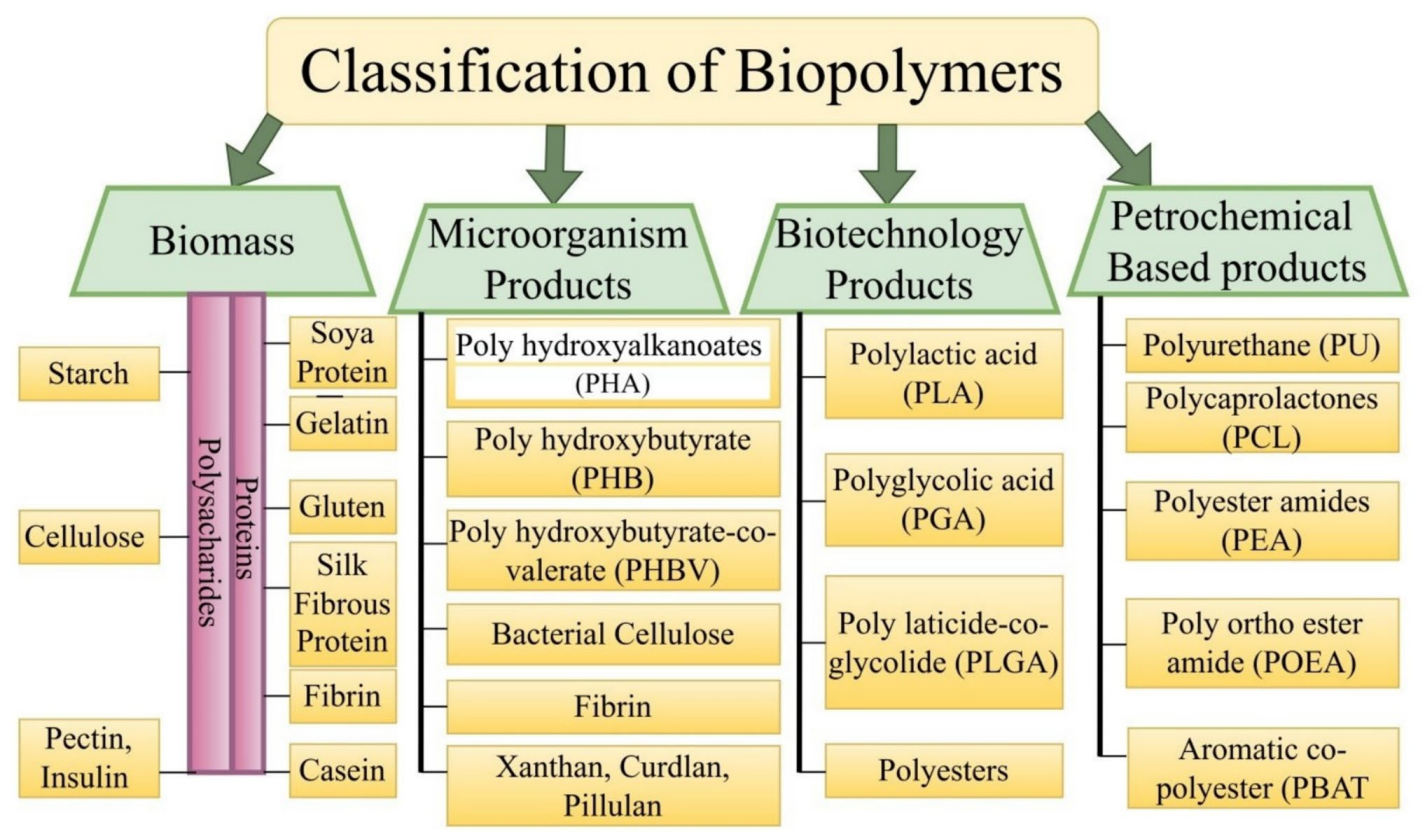
Classifications of biopolymers

Biodegradable polymers can be categorized according to their source, biological composition, and other characteristics. Biodegradable polymers can be naturally occurring or manufactured. Natural polymers are generated from naturally occurring resources such as plants, animals, and microbes, whereas artificial ones are chemically manufactured from hydrocarbons. The different functional groups include polyesters, polyamides, and polyurethanes. Figure 5 gives the classification of the biopolymers.

#### Biomass products

Starch and other biobased polymer compounds are naturally reintroduced as renewable polymers. The recovery and recycling of fossil-based polymers is the primary focus of efforts. However, because of end-of-life possibilities for fossil-based polymers, there is growing interest in replacing them with more environmentally friendly biopolymers.

##### Polysaccharides

The majority of biopolymers are polysaccharides, which contribute a significant character to the survival of living creatures. They have both environmental and multipurpose qualities. Starch, cellulose, proteins, and other similar terms are commonly used. Polysaccharides are made up of several monosaccharides, mainly C, H_2_, and O_2_. A monosaccharide element typically has 5–6 carbon atoms. Because the monosaccharide contains many hydroxyl groups (OH), H bonds are frequently encountered between polymer chains (Shang et al. 2013).

##### Starch

Starch is a particularly attractive agricultural biopolymer due to its widespread availability, low cost, and thermoplastic characteristics. Nonetheless, these polymers have substantial limitations, such as their hydrophilicity, difficulty handling, and poor mechanical performance, rendering them unsuitable for a wide range of applications (Moriana et al. 2011). Nonetheless, natural starch is more expensive than many synthetic polymers, limiting its application. A wide range of natural sources can provide plenty of starch. Starch granules come in many forms and sizes, ranging from 0.5 to 175 μm. Starch is made up of repeated elements of homoglucan or α-d-glucopyranosyl, which can produce amylose or amylopectin, and characteristics are determined by the molecular weight and branching grade of amylose and amylopectin (Elfaleh et al. 2023). Organic starches are semi-crystalline (crystallinity 20–45%) (Gurunathan et al. 2015). Dry starch density ranges from 1.514 to 1.520 g/cm^3^ while balance moistness content density ranges from 1.4682 to 1.4851 g/cm^3^.

#### Micro-organism products

Polyhydroxyalkanoate (PHA) and polyhydroxyalkanoates are hydroxyalkanoate (HA) polyesters obtained from a variety of naturally occurring sources including renewable sources (such as starch and cellulose), by-products (such as molasses), and CO_2_ (Al Hosni et al. 2019). In PHA, an ester link is formed by the carboxyl and hydroxyl groups of neighboring monomers. Several researchers have testified to the enormous manufacturing of bacterial PHA for industrial purposes. These substances are significantly more costly to process than fossil-based polymers. Approximately 150 varieties of HA monomers with diverse physical and chemical properties have been identified based on their geometry and side chain composition (Park et al. 2017). PHA copolymers such as scl-PHA (poly(3-hydroxybutyrate-co-3-hydroxyvalerate (PHBV)), mcl-PHA (poly(3-hydroxyhexanoate-co-3-hydroxyoctanoate) P(3HHx-co-3HO), and copolymers of scl and mcl PHA monomers (lcl-PHA, 2 bonds on the left-hand side C14) have mechanical characteristics similar to polypropylene and polystyrene (Westlie et al. 2022). PHAs can be eliminated in animal tissues by both non-enzymatic and enzymatic hydrolysis by microorganisms. The biocompatibility of materials is influenced by stereoregularity, chemical composition, inner morphology, molecular mass, and surface area; additionally, environmental conditions such as temperature, humidity, and pH affect disintegration capabilities.

#### Biotechnology products

Polylactic acid is an extremely utilized thermoplastic polyester with an opportunity to replace older fossil-based polymers. PLA is synthesized using lactic acid polymerization or polycondensation (Murariu and Dubois 2016). Crop residue fermentation allows for the larger-scale processing of lactic acid monomers. Lactic acid is available in a variety of stereochemical forms, including poly (d-lactic acid) (PDLA) and poly (l-lactic acid) (PLLA) (Nofar et al. 2019). Numerous studies have looked into the biodegradability degree of PLA (Vink et al. 2003). Temperature, time, and the concentration of the catalyst all significantly impact PLA disintegration rates. Oligomers and catalysts reduce disintegration temperatures while increasing disintegration rates. PLA's unique characteristics make it helpful in a wide range of industries, notably car components, textiles, tissue engineering, food packaging, and others. However, various fundamental shortcomings, such as inherent brittleness, poor energy absorption, delayed degradability, and so on, limit its use in a number of industries. Several studies were conducted to examine environmental resilience using life cycle evaluations.

#### Petrochemical based products

Polyurethanes are polymeric structures which are prepared by using different forms of urethane. Polyurethanes can be linked linearly or branched since they are made by polyaddition of a polyol and a polyisocyanate. Furthermore, polyurethane can be made by reacting diisocyanates with diols that have a chemical structure comparable to nylon (RG et al., 2023). Polyurethanes are chemically divided into four types: polyester, polyether, polysiloxane, and polycarbonate urethanes. Polyester urethanes are quickly biodegraded by microbial infection because they contain glucose, fructose, and sucrose. Polyurethanes are utilized for a variety of applications, including adhesives, insulators, and car parts. Polyurethanes have lately achieved favor in tissue engineering and medicine due to their outstanding energy absorption, flexibility, and deformation stability. Polyurethane has little moisture resistance and a low melting point (Mokhothu and John 2015).

### Properties of biopolymers

**Fig. 6:**
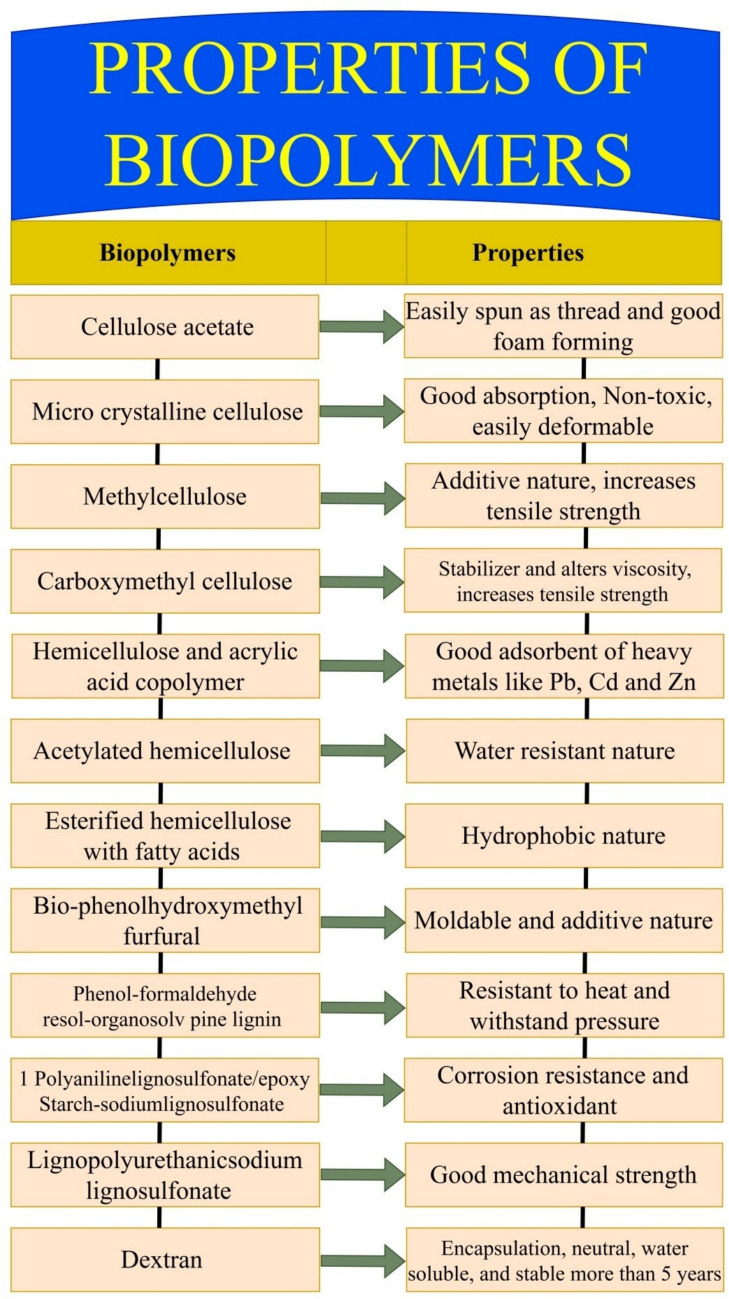
Characteristics of biopolymers

Polymers which are extracted from natural resources and biodegradable in nature are considered as biopolymers. The common natural resources for biopolymers include plants, animals, and microorganisms (Yusoff et al. 2016). Biopolymers are associated with various properties, as shown in Fig.6, which are generally useful to ensure various structural applications. Polymers are developed through organic materials, making the qualification biodegradability property an important parameter. It must be biocompatible with living tissue, and it does not exhibit an adverse response against immunity (Jana et al. 2021). As biopolymer-based products are used for structural applications, the product should exhibit mechanical characteristics such as strength, stiffness, and elasticity to qualify for structural applications (Anju et al. 2020). A few properties like melting point, thermal permanency, and transition temperature should be properly addressed for particular biopolymers (Arif et al. 2019). Moreover, hydrophilic, hydrophobic, solubility nature, rate of degradation, non-toxicity, material transparency, antimicrobial action, adhesiveness, and gel-forming capacities are important properties related to biobased polymers (Bernard et al. 2018).

The responsible factors such as the mixing process of biopolymer utilized plasticizer and reinforcement can upset the physicochemical and thermo-mechanical properties of biopolymers. The chemical structure and composition, functional groups, and molecular weight of biopolymers have an impact on their mechanical properties and elasticity (Fekete et al. 2019). TPS is an amorphous, semi-crystalline substance composed of gelatinized starch and one or more plasticizers. PLA polymers can range from amorphous glassy polymers having a glass transition temperature of 60 °C to semi-crystalline/highly crystalline products with melting points ranging from 130 to 180 °C (Mohammad et al. 2019). PLA offers low melt flexibility, which causes a variety of issues, including significant necking and deprived bubble constancy throughout extrusion, film casting, and blown film manufacture (Hassan et al. 2019). It has an excellent flexural modulus, is resistant to fatty foods and dairy products, heat sealable, and has a strong surface, which makes it suitable for printing and also possesses an exceptional polish and optical clearness (Tudorachi et al. 2015). Hydroxyethyl cellulose is a biopolymer with high temperature tolerance, salt resistance, and stability in neutral/basic pH solutions. Methylcellulose is highly thermally stable and water soluble and can produce gel by breaking hydrogen bonds between the polymer and its surrounding environment (Machado et al. 2020). PLA has higher flexural rigidity than polystyrene and is less susceptible to milk products and fatty meals than poly (ethylene terephthalate). It has a pleasant aroma and flavor in food packaging and has an outstanding surface toughness that enables easy 3D printing (Oksman et al. 2003). Amorphous and biaxial PLA films have high gloss and transparency comparable to PET and orientated PP (Andrew and Dhakal 2022). Biopolymer-based materials are appealing choices for tissue engineering since they break down or disintegrate in the host body with no negative repercussions. Biopolymers can be manipulated to change their properties for a range of applications, such as pharmaceutical administration, packaging, and electronic uses (Rostami et al. 2019).

### Advantages and limitations of biopolymers

The biopolymers have some advantages as well as limitations, as given in Table 3. Due to the use of natural resources, the biopolymers are favorable to the environment. In order to develop biopolymer matrices, there are various natural materials available in the environment. These materials can include plants, animals, and micro-organisms (Hernández et al. 2014). These can be available sources that contain proteins, polysaccharides, and nucleic acids. A variety of sources can be utilized to develop biobased polymers for various structural applications (Mtibe et al. 2021). These biobased polymers possess various advantages, such as biodegradability, sustainability, lower carbon footprint, non-toxic nature, versatility, and the ability to naturally decompose due to the action of microbes (Heinrich 2019; Cywar et al. 2022). This provides options to reproduce biopolymers with the help of renewable natural resources, which can reduce the depletion of conventional resources (Hottle et al. 2013). As conventional natural resources are responsible for generating a carbon footprint, as a result, the carbon footprint can also be reduced by using biopolymers (Stoica et al. 2022). The biopolymers generally used for the purpose of medical applications (polylactic acid and chitosan) are non-toxic and can be suitable for wound dressing and implants.

**Table 3:** Advantages and limitations of biopolymers

S. No.	Biopolymers	Advantages	Limitations	Sources
1	Polyhydroxyalkanoates (PHA)	Biodegradable, biocompatible, implant-friendly, derived from industrial and ecological by-products	PHA application is limited by poor mechanical properties, high production cost, and limited microbial sources	(Liu et al. 2021)
2	Polylactic acid (PLA)	Biodegradability, high mechanical strength, biocompatibility, resistance to heat and water solubility, ideal packaging material	Insufficient creep behavior, limited mechanical and barrier performance, susceptible to thermal deformation	(Swetha et al. 2023)
3	Polyvinyl acetate (PVA)	Better tensile strength, adhesion to hydrophilic surfaces, high transparency, and stability	High swelling, compression, Ionic permeability, low flux at high crosslinking, and close melting and aging temperatures, high water solubility	(Deng et al. 2024)
4	Polycaprolactone (PCL)	Excellent viscoelastic, rheological, and mechanical properties. Biocompatible, less inflammability, low cost	Low cell adhesion, Slow degradation	(Arif et al. 2022)
5	Styrene/high impact polystyrene (HIPS)	Impact resistance, high rigidity and good moldability, opaque	Low heat resistance, brittle at low temperature	(Mohan et al. 2016)
6	Polyglycolic acid (PGA)	Biodegradability, good tensile strength, biocompatible, controlled degradation, low permeability	Fast degradation, lost mechanical properties after degradation, brittleness, high cost	(Soleymani et al. 2024)
7	Polylactic and glycolic acid (PLGA)	Biodegradable, manageable degradation rate, biocompatible, applicable in biomedical implants	Limited mechanical strength, brittle, hydrolytic degradation, produces acidic by-products, complex production techniques	(Jin et al. 2021)
8	Polybutylene succinate (PBS)	Biodegradability, cost effective, good mechanical properties, thermally stable, high process ability	Low stiffness and tensile strength, brittle at low temperature, low melting temp., high moisture absorption	(Barletta et al. 2022)
9	Polybutylene adipate-co- terephthalate (PBAT)	Enhance the films’ mechanical performance, transparency, and seal ability, flexibility and stiffness, compatibility, high thermal stability ~400 °C	Slow degradation rate, low heat resistance, poor UV resistance, and limited structural application	(Roy et al. 2024)

3D printing is an important step in the production of biobased composites. There are multiple advantages of biodegradable polymers produced with the help of 3D printing (Cakir Yigit and Karagoz 2023). These advantages are consistent with both environmental and technological factors. In the beginning, artificial polymers derived from renewable sources such as cornflower and sugarcane considerably decrease the dependence on fossil fuels (Sadeghi et al. 2020; Calvo-Flores and Martin-Martinez 2022). Moreover, biodegradable plastics have a lesser environmental impact because they degrade naturally over time, reducing long-term waste (Lagman-Bautista 2020; Silva et al. 2023). In regard to 3D printing technological advances, these materials are preferable. There are various advantages to 3D printing, including easiness of use and adaptability, which allow for complicated designs and precise prints (Faidallah et al. 2022; Randhawa et al. 2023). Furthermore, 3D-printed biodegradable polymers are compatible with a broader spectrum of 3D printers. As a result, 3D printing machinery expands the use of biodegradable polymers (Dananjaya et al. 2024). Overall, using biodegradable polymers in 3D printing enhances sustainability, simultaneously extending the availability of numerous innovative manufacturing solutions.

The various factors restrict the utilization of biobased polymers to their full potential. The production cost usually hampers the amount of manufacturing of biobased polymers, which could not be cost-effective in terms of raw materials and biotechnological procedures (Ravula et al. 2019). Most of the biopolymers are less effective in terms of durability, strength, and flexibility. These limitations generally restrict the biobased composite materials in high-performance structural applications, such as polylactic acid (PLA), which is brittle in nature and has lower durability (Tardy et al. 2017). Some of the biodegradable polymers can have their use restricted in open environments due to adverse environmental impacts such as moisture, heat, and ultraviolet radiation, which limits the applications for long periods and high-stress applications (Vinod et al. 2020). The biobased polymer development technology is new as compared to traditional polymer technology and needs the development of new infrastructures. Agro-waste is a valuable source of biopolymers, which raises ethical anxiety related to food safety. Biopolymers that are made up of food grains can hamper food needs.

## Biodegradable polymeric composites

Composite products, combinations, and hybrid substances are promoting sustainable manufacturing advancement in the field of 3D-printed biodegradable polymers (Singh et al. 2020). As indicated in Table 4, these materials combine the inherent environmental friendliness of biodegradable polymers with enhanced characteristics and a greater range of uses. For example, in composites, natural fibers or nanomaterial’s are utilized to reinforce a biodegradable polymer matrix (Mencik et al. 2019; Wang et al. 2017). The materials produced by this process have increased strength, durability, and environmental responsibility, making them suitable for implementation in the construction, aerospace, and consumer goods industries (Chohan et al. 2022). The capacity to accurately regulate the composition of these composites permits the formation of unique solutions that flawlessly balance performance and sustainability. Conversely, blends entail the deliberate blending of various biodegradable polymers (Wissamitanan et al. 2020). Manufacturers can produce materials with specific properties by combining different polymers, striking a careful balance between strength, biodegradability, and other desired qualities (Govindan et al. 2023). Because of their flexibility, biodegradable materials may be produced to meet a variety of industrial needs such as those for flexible packaging (Azizoğlu and Özer 2020), farming applications (Ju et al. 2022), and medical-based equipment’s (Gelinsky 2017). Those biodegradable polymer combinations are bringing in a new era of sustainable resource innovation, giving environmentally responsible options while maintaining effectiveness.

**Table 4:** Properties of 3D-printed biocomposite

S. No.	Matrix	Filler	3D printing method	Remarks	Sources
1	PLA	Poplar wood	Fused deposition modeling (FDM)	The torque increases to 7 N-m whereas impact strength reduces to 1.5 kj/m^3^ at 10 wt.% filler	(Zhang et al. 2018b)
2	PLA	Lignin	Fused filament fabrication (FFF)	The tensile strength to 0.5 MPa from 0 to 5 wt.% loading	(Gkartzou et al. 2017)
3	PLA	Hemp and harakeke	FDM	The tensile strength decreases by 31.42% at 30 wt.% while Young’s modulus increases by 42.5% at 20 wt.%	(Stoof et al. 2017)
4	PCL	Cocoa shell waste (CSW)	FDM	Young’s modulus increases to 52 MPa at 30 wt. % CSW	(Tran et al. 2017)
5	PLA	Anchovy fishbone powder	FDM	Flexural modulus improves by 23%	(Scaffaro et al. 2022)
6	PLA	CaCO_3_	FDM	Compressive strength improves to 50 MPa	(Qin et al. 2022)
7	Bioplastic + PLA blend	Carbon from waste coconut shell	FDM	Tensile strength increases by 50% at 0.6 wt.% of carbon	(Umerah et al. 2020)
8	PLA	Ceramic waste	FFF	The flexural strength decreases to 2.5 GPa at 10 wt. % of ceramic waste	(Fico et al. 2022)
9	PHA	Palm fiber	FDM	Tensile stress is decrease from 16.7 MPa to 9.6 MPa up to 20 wt.% palm fiber loading	(Wu et al. 2017)
10	PCL	Graphene oxide	FDM	Improved the tensile strength, modulus, and energy at PCL membrane by 95%, 66%, and 416%	(Wan and Chen 2011)

**Fig. 7:**
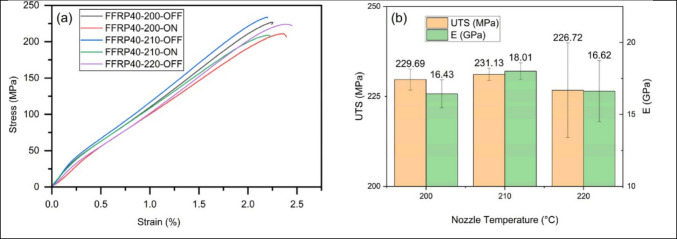
**a** The tensile curves of the different configurations and **b** tensile strength and modulus as a function of the nozzle temperature with the FAN off

**Fig. 8:**
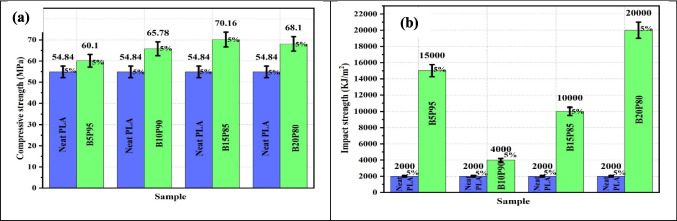
Plot for mechanical properties of the composites. **a** compressive strength and **b** flexural strength

**Fig. 9:**
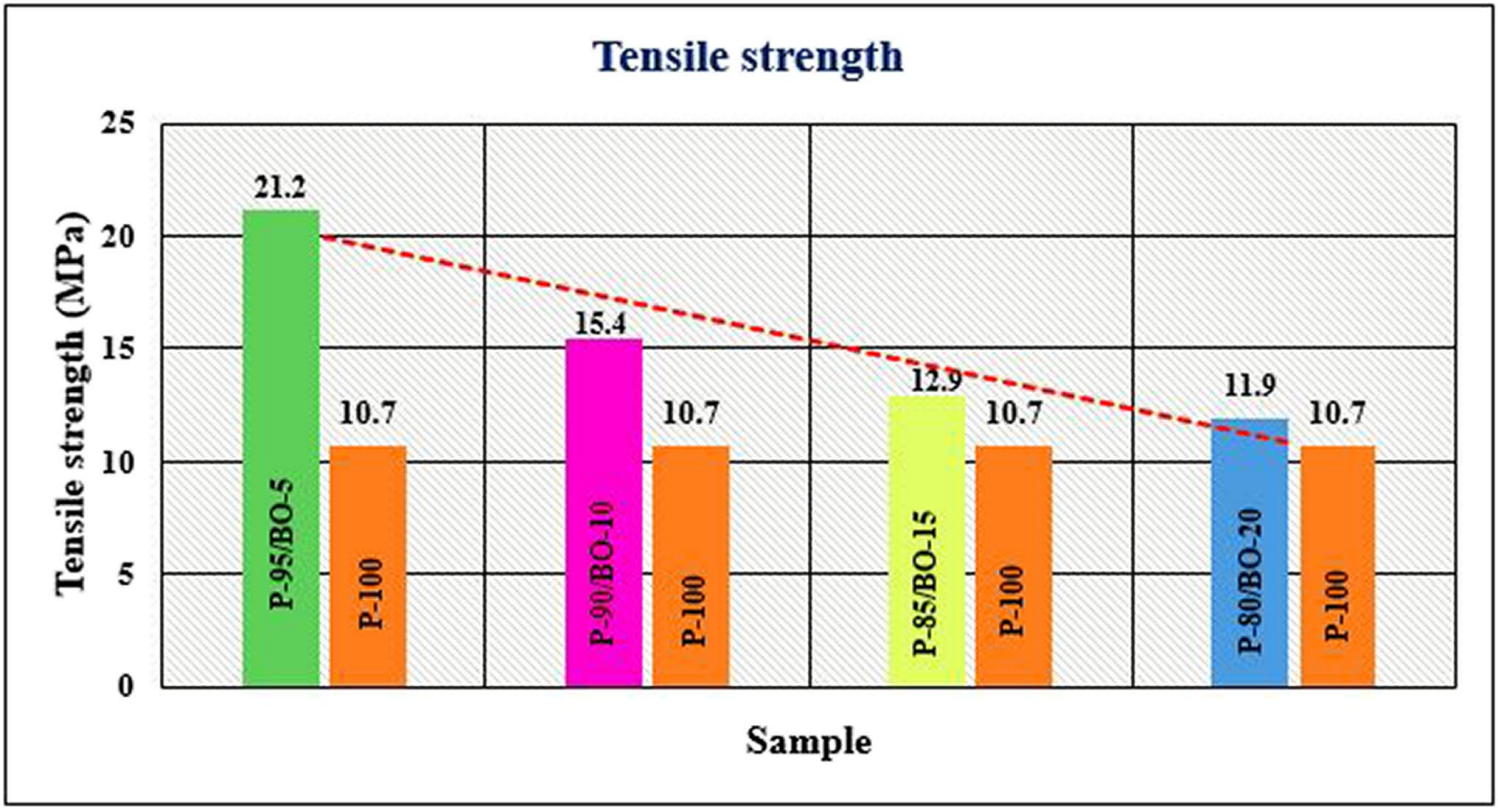
Tensile strength of the biocomposites

The design of hybrid materials for 3D-printed biodegradable polymers is a growing field. Biodegradable polymers can sometimes be combined with non-polymeric materials such as metals or ceramics utilizing advanced methodologies for 3D printing (Kim et al., 2023). These combinations integrate the benefits of biodegradable polymers along with the environmentally friendly properties of metals or ceramics such as electrical conductivity or heat resistance (Xiong et al. 2020). This opens up a widespread domain of applications containing strong but lightweight structural elements, sustainable medical implants, and ecologically friendly electronic components (Bastola et al. 2018). As investigation and improvement into these disciplines endure, the promise for a more sustainable and ecologically friendly future driven by advanced 3D-printed biodegradable materials becomes increasingly attractive. The sustainable starch-based packing wastes have been utilized in the development of high-quality biochar. The modified biochar has been successfully reinforced with polypropylene in composites, which have demonstrated an improvement in tensile strength by 46 wt.% with the reinforcement of 0.74 wt.% of biochar (Mohammed et al., 2022). The evaluations of the optimal printing parameters for the different fractions of the flax filament have found an improvement in the tensile fractions as shown in Fig. 7a and b; also, the tensile properties are influenced by the damage mechanism (Mehaba et al., 2025). The work has demonstrated the impact of industrial forest residue including cellulose fibers, wood ashes, and jack pine sawdust and found a reduction in the degradation of PLA and glass transition temperature, whereas the crystallinity was improved (Helaoui et al., 2024). The banana peel powder (BPP) was successfully incorporated with Polylactic acid (PLA) in biocomposites and found that the mechanical properties of the biocomposites improve with the reinforcement of BPP, as shown in Fig. 8a and b (Soni et al., 2025a). The hybrid fruit waste-derived biofillers of BPP and orange peel powder (OPP) were utilized in the development of PLA-based biocomposites and found a significant improvement in the properties as given in Fig. 9 (Soni et al., 2025b).

### Characterizations of 3D-printed biocomposites

The different characterization techniques are crucial to assess 3D-printed biocomposites’ structure, quality, performance, and applicability for specific applications. The techniques are broadly classified as physical, mechanical, thermal, chemical, and morphological analyses. The bulk characteristics of 3D-printed biocomposites that determine their functional applicability are revealed by physical characterization. The measurement of density and porosity is essential because it shows how voids form during printing, which has a direct impact on durability and mechanical strength. Adhesion, wear resistance, and interactions with the environment are all significantly influenced by the surface properties of 3D-printed biocomposites. The dispersion of natural fiber, raster orientation, and filament deposition all contribute to surface roughness, a prevalent characteristic in fused deposition modeling (FDM). The work has demonstrated the effect of surface characteristics such as roughness and peak-to-valley height on the wetting behavior of the specimens by the contact angle technique. The 3D-printed specimens were fabricated by using four different printing layers of 0.05, 0.1, 0.2, and 0.3 (mm) in the production of the 3D-printed specimens. The smoothness of the specimen improves with the decrease in printing layers, whereas the wettability improves with the thickness of the printing layer (Ayrilmis 2018).

The mechanical properties of the 3D-printed composites are influenced by the properties of the raw materials and the type of manufacturing techniques (Alarifi 2024). The materials with defined functions are considered for 3D printing, and reinforcements are incorporated to improve the properties of the composites (Li et al. 2023). The layer processing of the polymeric materials has several issues limiting their applications. The issues must be addressed suitably to broaden the application of 3D printing. The mechanical anisotropy has created an issue in additive manufacturing. The work has studied the effect of the size of the layer; consequently, thickness, raster angle, air gap, orientation of filament, and build direction in the development of 3D-printed biocomposites (Iyer & Keles 2022; Mathiazhagan et al. 2023; Meddeb et al. 2024). A well-designed 3D-printed object should have minimum anisotropy and outstanding quality. The load and strains in a 3D-printed item should be aligned with the material's strongest orientation. If raw materials with adjustable mechanical qualities become accessible, these may be acquired. The researchers have generally accepted ASTM standards for conducting their mechanical tests. For instance, the researchers have followed ASTM D638 for the assessment of tensile tests (Appalsamy et al. 2024; Karad et al. 2023). However, because of issues with sample geometry in the ASTM D638 standard which tends to cause the sample to fail early, particularly at the radiused corners, some groups choose to follow ASTM D3039 (Bendine et al. 2022; Yıldız et al. 2022). Due to weak interlayer bonding, samples loaded along the build direction have the worst tensile properties, while filaments oriented longitudinally and parallel to the direction of loading have the best tensile properties (Gajjar et al. 2025). The PLA sample's ultimate strength was the highest for the 45° raster angle compared to the 0° and 90° raster angles (Ayatollahi et al. 2020), the PEEK sample’s greatest strength was for the 0° raster angle (Xu et al. 2022), and the ABS sample’s greatest ultimate strength was for the 0° raster angle compared to the 45° and 90° raster angles (Nabavi-Kivi et al. 2023). Because space applications, where temperatures in the International Space Station vary from −157 to 121 °C, are potentially a possibility in the near future, it is particularly crucial to comprehend the mechanical behavior of 3D-printed parts at cryogenic temperatures. Young’s modulus at the cryogenic temperatures was greater than that at room temperature when the tensile characteristics of ABS at ambient temperature, 77 K (liquid nitrogen temperature), and 4.2 K (liquid helium temperature) were examined. However, as compared to the sample tested at normal temperature, the ultimate tensile strength at cryogenic temperatures was somewhat lower (Vaught et al. 2023).

In order to assess the stability, process ability, and long-term performance of 3D-printed biocomposites under service settings, thermal characterization is crucial. The inherent characteristics of both phases and interfacial bonding produced during additive manufacturing have a significant impact on the thermal response of biocomposites because they blend natural fibers or fillers with thermoplastic or thermoset matrices. Thermogravimetric Analysis (TGA) is frequently used to evaluate the degradation behavior and thermal stability of biocomposites that are 3D printed (Ali et al. 2023). It offers details on the temperature at which degradation begins, the char yield, and weight loss patterns related to the breakdown of cellulose or lignin, hemicellulose, and the polymer matrix. Because lignocellulosic fibers have a lower decomposition temperature, the use of natural fillers frequently results in decreased thermal stability (Awad et al. 2023). Thermal resistance can be improved considerably through suitable surface treatments or hybrid reinforcements (Krishnasamy et al. 2019). The glass transition temperature (Tg), crystallization behavior, and melting properties of polymer matrices augmented with biobased fillers can be studied with the use of Differential Scanning Calorimetry (DSC). Fibers have the ability to adjust temperature transitions and change crystallinity levels by acting as nucleating agents. In general, higher crystallinity enhances mechanical performance and thermal stability; however, excessive filler loading may limit chain mobility, which lowers crystallization efficiency (Ming et al. 2022).

## Manufacturing of biocomposites

The fabrication of biocomposites is defined as the transformation of raw materials into composites of desired shape, properties, and size. The behavior of the biocomposites is influenced by the conditions and methods of manufacturing (Faruk et al. 2012). In general, the manufacturing techniques for conventional and biocomposites are not very different. The conventional methods, including molding, extrusion, compression, etc., can successfully develop thermoplastic-based biocomposites (Manral et al. 2021). The selection of an appropriate procedure for the production of biocomposites depends on the fabrication cost, handling qualities, quantity of fabrication, characteristics, and size of composites.

### Conventional fabrication methods for biocomposites

The conventional technique used for the development of synthetic composites can be successfully implemented for the development of biocomposites. Table 5 summarizes the benefits and limitations of the various production strategies used to fabricate biocomposites. The available methods for the development of composites are well-developed and can successfully develop composites with the required characteristics and quality. In order to acquire high-performance biocomposites for different applications, there is an urge for the exploration of advanced technologies and solutions.

**Table 5:** Benefits and limitations of biocomposite manufacturing techniques

S. No.	Technique	Advantages	Limitations
1	Wet/hand lay-up	Simplicity, low cost, feasibility to develop any combination of material	High voids, requirement of less viscous resin, consideration for safety and hazard, distribution of resin effects the quality
2	Spray lay-up	Economic, low tool cost and material systems, suitable for wide range of products	Limited to short fibers; unsuitable for structural demands; challenges in fiber control, repeatability, and accuracy; operator-dependent; requires low-viscosity resin; emits styrene; uneven surface finish
3	Autoclave curing	Ensures better resin distribution, large component fabrication, good surface finish, and minimal contamination risk	Require expensive tool, high operation and maintenance cost. Unsuitable for small components
4	Filament winding	The process is rapid, Economic and better load bearing structures	Requires low viscosity of resin, restricted convex form parts, high Mandrel prices, low surface finish
5	Pultrusion	Suitable for large-scale, fast, and cost-effective production with precise resin control, low fiber cost, good surface finish, and strong properties	Limited to constant cross-sections; costly heated die; unsuitable for narrow profiles

### Advanced fabrication techniques

The majority of traditional composite production techniques need moulds, which restrict formability and make the manufacturing process time-consuming and expensive. Additive manufacturing, also known as 3D printing, is a method of production in which elements are built up layer by layer using computer-assisted design tools (Liu et al. 2019; Khalid et al. 2023). In recent years, the additive manufacturing process has been increasingly used for the production of superior lightweight materials and structures. The fabrication of biocomposites via additive manufacturing includes primarily three steps: (i) in this process, materials are ejected against a construction plate using a heated nozzle, (ii) Vat photopolymerization employs the curing photopolymer by using ultraviolet for fabrication, and (iii) power bed fusion fuses the powder bed by utilizing heat energy. Considering the several manufacturing techniques, fused filament fabrication (FFF) is the frequently utilized for the fabrication of biocomposites. The profits of FFF include less production charge, less time taking, and the potential to develop complex components with minimum waste (Rajak et al. 2019). The printing parameters, such as interbreed distance, print orientation, layer thickness, etc., and the slicing parameters, including filament feed rate, nose geometry, bed temperature, etc., influence the mechanical characteristics of biocomposites (Tofail et al. 2018). The AM techniques have gained prospects to fill the gap with synthetic composites and are favoring the technological advancement of biocomposites. The different polymer-based composites have been successfully employed by incorporating reinforcements with fillers and fibers in polymers. The reinforcement of filler with the polymer matrix is simple and cheap; thereby, the fabrication of FFF and stereolithography (SLS) is comparatively easy. The resulting material shows an enhancement in the electrical, tribological, thermal, and biological characteristics. However, the agglomeration of the nano-fillers is creating an issue in uniform distribution. Various multi-structured substances with specialised qualities have been produced using stereolithography (SLA) (Vitale et al. 2015). The fabrication and development of polymer-based biocomposites incorporated with nano-fillers open avenues in composite manufacturing. The strength, interfacial connections, and mechanical characteristics of the SLA substrate can be efficiently enhanced by treating the nanofiller with silane or thermal treatment (Ligon-Auer et al. 2016). Shorter and continuous fiber reinforcement may enhance the characteristics of SLA resin-based polymeric composites (Parandoush and Lin 2017). FFF, like particle-reinforced raw materials, is an extremely often-used AM process for producing composite materials.

## Factors affecting the development of biocomposites

The lower strength of biocomposites is a challenge in the fabrication of biocomposites. Therefore, the pretreatment of the biocomposites is an important step to advance the properties of the composites. Features such as type and characteristics of reinforcement, distribution of reinforcement, hydrophilicity, content, and machining are the important factors that must be controlled in the development of biocomposites. This section discusses the different factors which influence the production of biocomposites.

### Moisture content and hydrophilicity

The amount of moisture has a significant impact on the characteristics of biocomposites; hence, dewatering the reinforcement is vital in the creation of biocomposites (Dhakal et al. 2007). In order to improve the efficiency of biocomposites, water absorption must be less than 3%. Furthermore, the material must be carefully treated and preserved to avoid a bursting of dust fragments. Moisture in the reinforcement can cause voids and water vapor in biocomposites during production. The biocomposites made with pennywort fibers have a high moisture content of approximately 57% and relative humidity of 90%, rendering them sensitive to microbial attack. The presence of hydrogen bonds that have powerful polar groups influences the moisture absorption of biocomposites in elevated and ambient environments. The relative humidity primarily affects the absorption rate and moisture content of the biocomposites (Stamboulis et al. 2000). A suitable chemical treatment increases the cellulose crystallinity and removes the hemicellulose, which reduces the moisture content of biomaterial-based reinforcement. The various procedures, including acetylation, copolymerization, coupling agents, and so on, enhance the fiber wetting capacity. Biofiber-based composites are more susceptible to moisture. The scholars investigated the effect of coupling agents on the moisture action of corncob, wheat straw, and cornstalk-based biocomposites, which found that the cornstalk and wheat straw composites experienced a reduction in moisture content as a result of the coupling agent, while the corncob-filled composites responded differently (Panthapulakkal and Sain 2007).

### Thermal stability

**Fig. 10:**
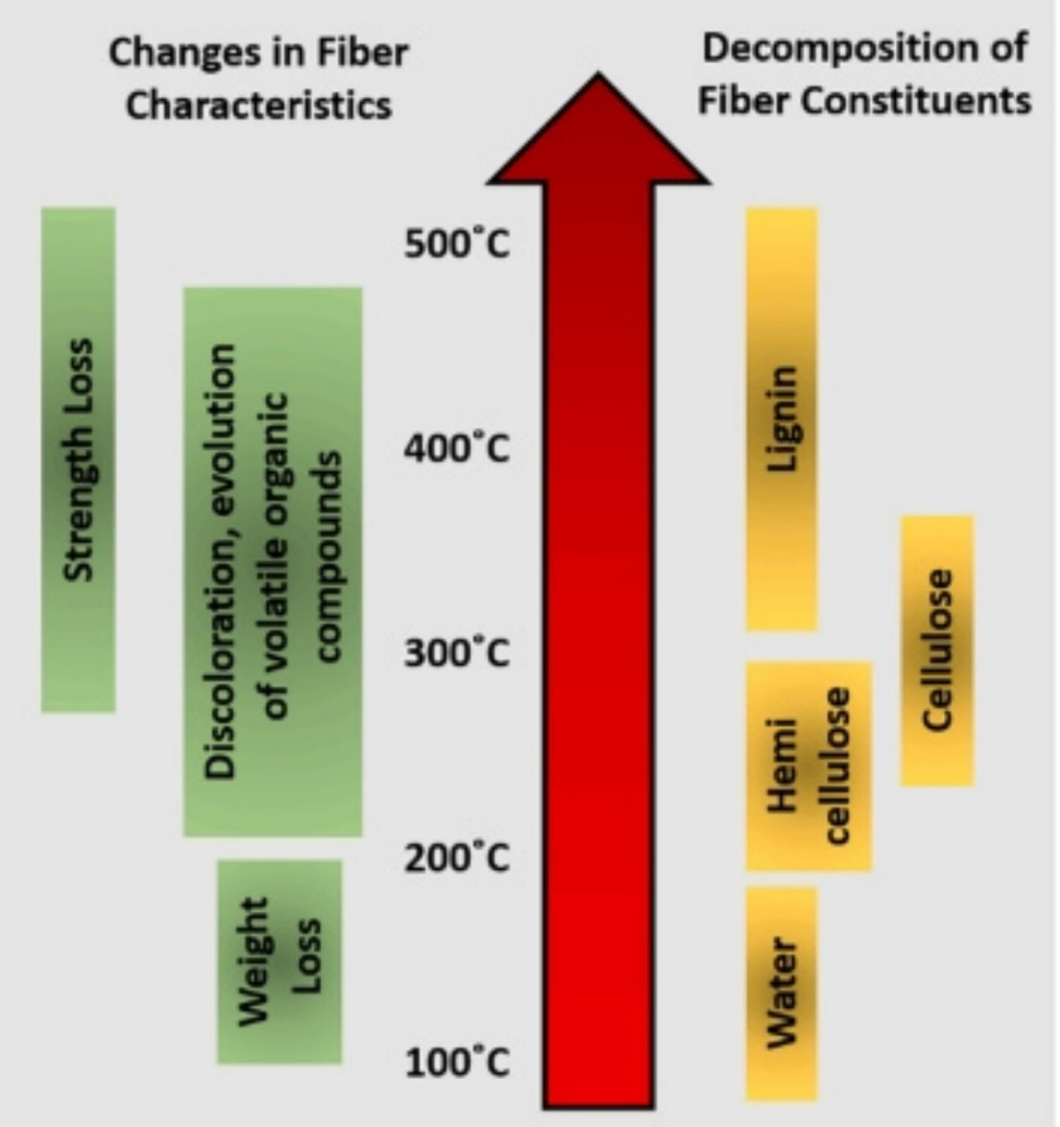
Change in fiber constituents at different degradation temperatures (Lau et al. 2018)

Biofiber breakdown accelerates as manufacturing temperature rises. The physical and chemical changes due to the heat induce the degradation of fibers (Poletto et al. 2012). The thermal integrity of biocomposites is determined by its moisture level and composition. The degradation of the compositions of fibers with temperature is demonstrated in Fig. 10. The degradation of the biofibers initiates with the degradation of hemicellulose (Ornaghi et al. 2014). Biofibers with high-quality extractives and hemicellulose are especially susceptible to thermal breakdown. Fibers with a high percentage of crystalline cellulose have excellent thermal resistance. Fibers can be physically and chemically treated to boost their resistance to heat in biocomposites.

### Fiber breakage

Fiber breaking during biocomposite manufacture is a challenge to achieve required mechanical properties. The extent of fiber degradation is largely determined by the manufacturing procedures used to generate biocomposites. Moreover, the different factors, such as temperature, pressure, fiber length, and shear rates, affect the breakage of fibers. The long processing temperature, high temperature, and rotor speeds lead to poor reinforcement and rupture of the fibers.

### Fiber type and content

The consideration for the reinforcement nature and their treatment is significant for enhancing the sustainability of biocomposites. Moreover, the aspect ratio and length of reinforcement have a considerable effect on the manufacturing parameters. The fibrillation, shortening, and thermal degradation of the reinforcement significantly affect the thermal degradation, fibrillation, and shortening of the reinforcement, whereas the commencement of the manufacturing method determines the characteristics of the final product (Yan et al. 2014). The strength and modulus of the composites improve with the volume fraction of the fibers, which increases the moisture content. Moreover, the dimension of the fiber plays a vital role in the manufacturing and processing of biocomposites (Gejo et al. 2010).

### Distribution of fibers

The processing of the biocomposites is influenced by the distribution of fibers. The improper or poor distribution of fibers results in cracking. The orientation and dimension of fiber, process parameters, and treatments influence the distribution of fibers in biocomposites (Serrano et al. 2013).

### Machining related challenges

The assembly of the biocomposite components is performed by machining. The complex microstructure of biocomposite makes the machining of biocomposite more complex as compared to synthetic and isotropic materials. Machining causes cracking, peel-up, deboning, and other damage, as well as a reduction in the mechanical properties of biocomposites (Sayam et al. 2022).

## 3D printing technique for biodegradable polymers

**Fig. 11:**
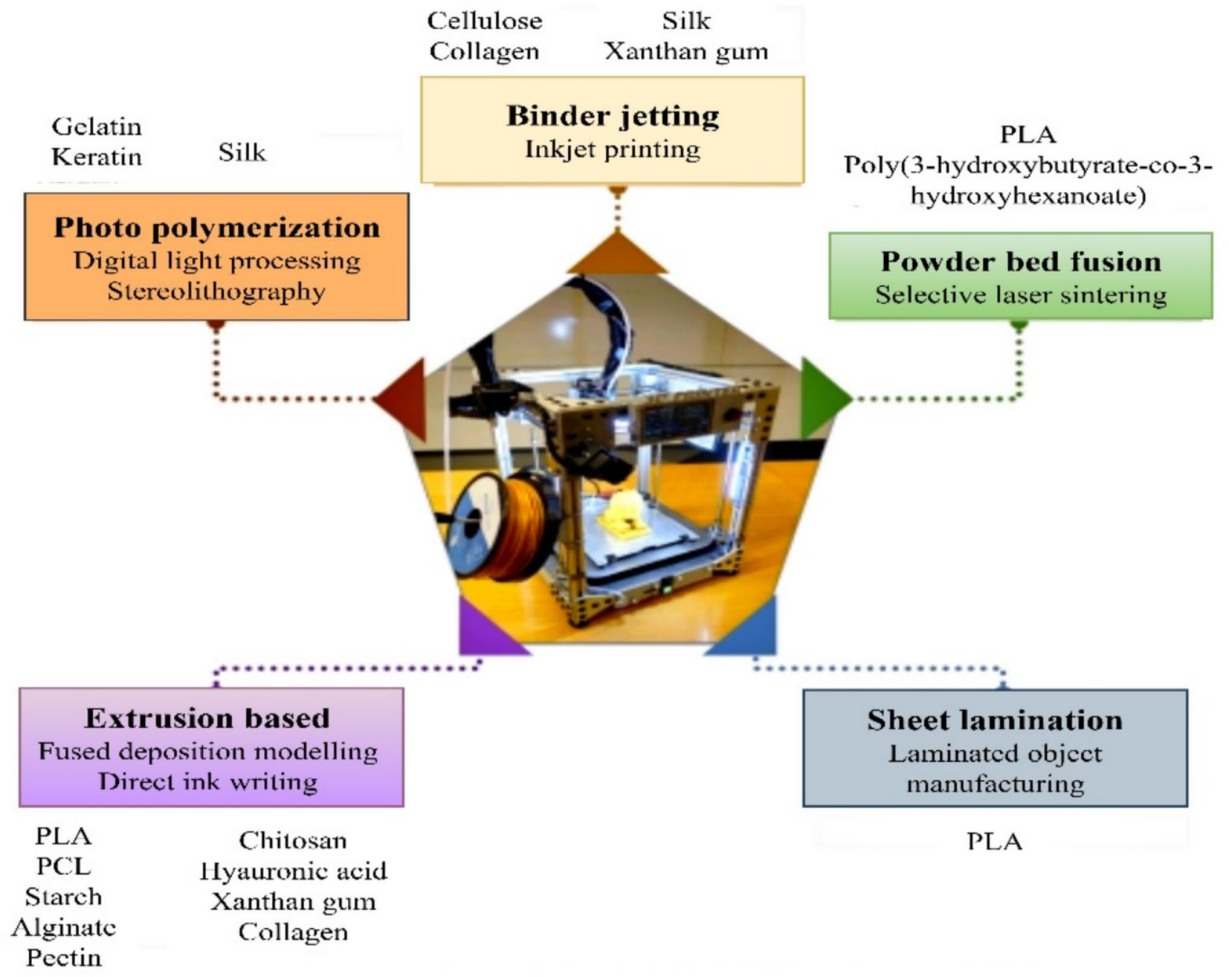
Additive manufacturing methods for biodegradable materials (Dananjaya et al. 2024)

3D-printed biodegradable polymers are an inventive and ecologically friendly industrial process. By using biodegradable materials, typically derived from natural sources like sugarcane, maize starch, or algae, this approach creates intricate and long-lasting 3-dimensional structures (Mazzanti et al. 2019). These polymers have the ability to degrade naturally over time, decreasing their adverse environmental effects and providing a remedy to the long-term issue of plastic waste. 3D printing allows for precise and adaptable manufacture, permitting the creation of biodegradable items ranging from packaging materials to medical implants. This unique strategy not only stimulates the creation of intricate designs, but it also aligns with the growing emphasis on sustainability and the circular economy throughout the world, paving the way for a more environmentally friendly manufacturing technique (Faidallah et al. 2022). The 3D printing of biodegradable polymers is given in Fig. 11.

**Fig. 12:**
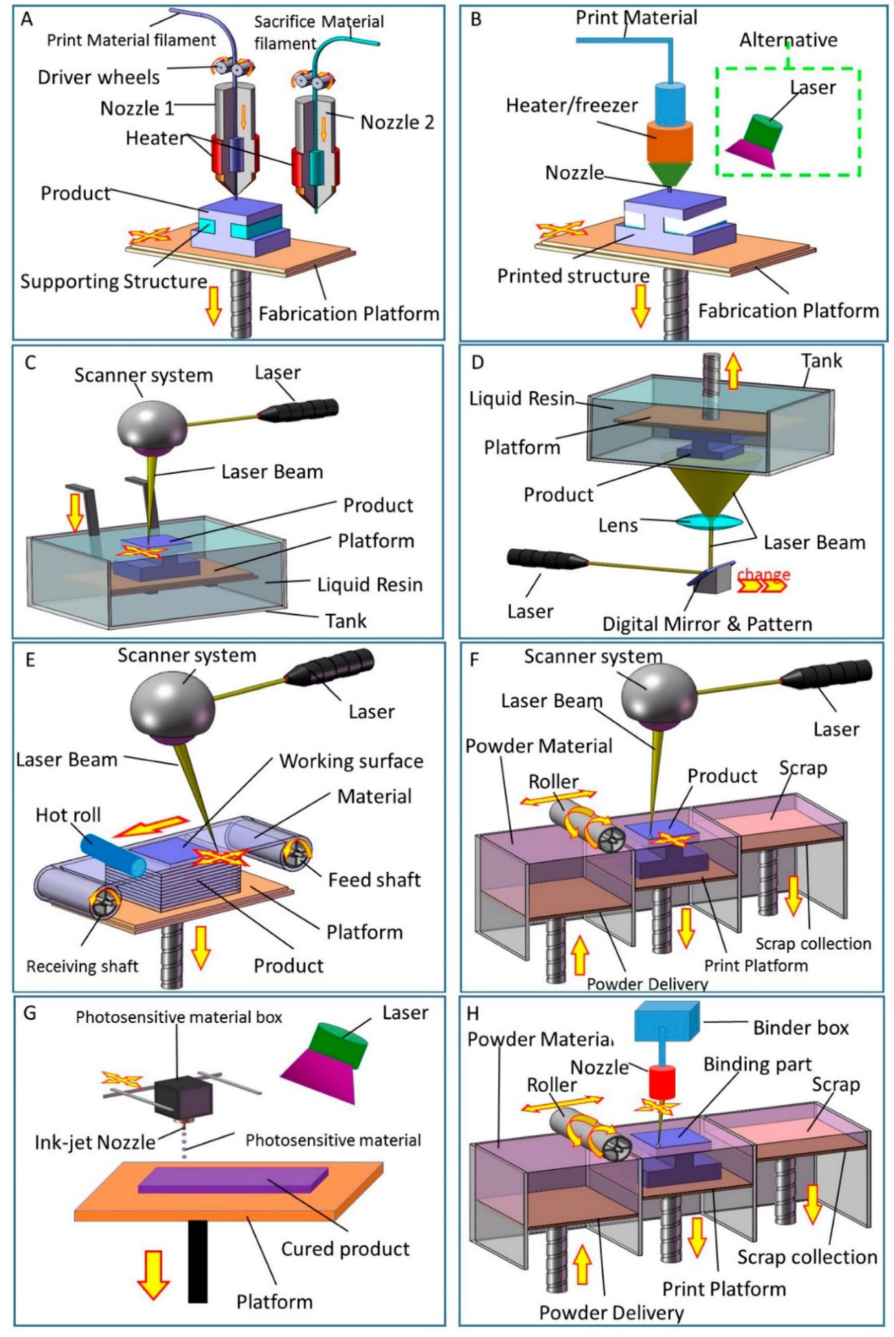
3D printing technologies. **A** FDM, **B** DIW, **C** SLA, **D** DLP, **E** lamination (LOM), **F** SLS and selective laser melting (SLM), **G** photopolymer jetting (Ployjet), and **H** Binder jetting (BJ) (Xu et al. 2017)

3D printing is based on a set of core concepts and methods for producing three-dimensional objects from digital blueprints (Perumal et al. 2021). All 3D printing techniques are based on the fundamental idea of building an object layer over layer by adding material, whereas the subtractive manufacturing methods involve taking material out of a bigger block (Baca and Ahmad 2020). The first step in the procedure for 3D printing is to create a digital 3D model using computer-aided design (CAD) software or by 3D scanning a real-world object (Mostafaei et al. 2021). Following that, cutting software is employed to divide it into thin cross-sectional layers. Typical 3D printing technologies include SLA, FDM, DLP, and selective laser sintering (SLS) as seen in Fig. 12 (Manapat et al. 2017; Niang et al. 2022; Klyosov 1986). Every technique takes a different approach to depositing, hardening, or fusing the components, producing a range of qualities and uses. To advance the features of the product and its functioning, post-processing procedures including surface polishing, support removal, and curing are necessary (Mostafaei et al. 2019). Plastics and other materials for building, such as metals and ceramics, are often used in 3D printing processes. Because of their adaptability and flexibility, 3D printing techniques have altered the production of goods (Exley et al. 2024; Yuan et al. 2020). It offers chances for complex design, quick prototyping, and personalization. Previously, the conventional production techniques were not capable of producing them. New ideas and methods will probably surface as technology develops, expanding the probable applications of additive manufacturing.

### Fused deposition modeling (FDM)

FDM is a highly popular and easily available 3D printing technology. Scott Crump invented FDM in the late 1980 s, which is the process of layer-by-layer extrusion and depositing thermoplastic materials to build multifaceted objects (Cano-Vicent et al. 2021). The procedure initiates with the feeding of a spool of filament, usually composed of Polyethylene Terephthalate Glycol (PETG), PLA, Acrylonitrile Butadiene Styrene (ABS), or nylon, into an extruder (Zhang et al. 2022; Fahad et al. 2023). On the basis of the digital 3D model, the extruder accurately places the heated filament onto the construction surface after warming it to the specified temperature. After completing one layer, the foundation drops and continues to add layers until the complete product is created. Since FDM produces accurate and durable parts, it is an excellent choice for end-use parts, functional prototypes, and projects. FDM is adaptable due to its wide choice of materials, simple use, and suitability for massive production. FDM has some downsides, including lesser resolution than other 3D printing techniques, visible layer lines, and the need for support structures when producing complex shapes or extensions (Woern et al. 2018a). However, the ongoing development and the introduction of twofold extrusion systems have increased its possibilities, securing its place as an adaptable and affordable 3D printing option (Parulski et al. 2021). Fused particle deposition is also a useful 3D printing method from a sustainability standpoint. The waste polymers can be put through the FDM process to develop the qualities of reprocessed products.

### Stereolithography (SLA)

The first 3D printing method in the history of additive manufacturing is known as SLA. It creates sophisticated, excellent quality 3D objects by photo polymerization (Huang et al. 2020). The method begins with a vat filled with liquid photopolymer resin, which is then selectively cured using an ultraviolet (UV) laser or other light source (Lakkala et al. 2023). The computerized 3D model shows how the UV laser scans the resin’s surface, hardening it layer by layer. After finalizing a layer, the build platform is dropped, and the procedure keeps happening until the entire 3D item has been developed (Khatri et al. 2020). In light of its popularity for creating smooth, detailed, complex geometries with fine features, SLA is an attractive option for applications such as the development of products, quick prototyping, and the fabrication of intricate models in a wide range of sectors (Karakurt et al. 2020). SLA has drawbacks when it comes to material choice and the requirement for post-curing to obtain the necessary mechanical qualities (Patmonoaji et al. 2022). However, ongoing developments in SLA technology, including better resins and quicker drying times, have increased its potential and opened up new avenues for 3D printing innovation. Numerous studies have used this additive manufacturing technique for biodegradable and non-biodegradable polymers (Nagarajan et al. 2019; Camenisch et al. 2025; Liu et al. 2018).

### Binder jetting (BJ) and laminated object manufacturing (LOM)

Binder jetting was initially established in the early 1990s. It is a flexible and potential 3D printing technique that creates complicated three-dimensional structures by deliberately layering a liquid adhesive onto a substrate of powdered material (Shakor et al. 2022). There are numerous benefits offered by this additive manufacturing technique to its customers. Among these are speed and cost-effectiveness, since they use less material and do not require expensive support structures as in conventional production techniques (Chen et al. 2022). After every single layer of powdered material has been thoroughly covered with the binder, a new layer of powder is distributed and the process is repeated until the entire item is formed (Salari et al. 2022). After printing, the green component is removed from the loose powder and undergoes a subsequent processing step, typically penetrating or sintering, to solidify and reinforce the object (Li et al. 2020a, b, c). In several industries, including the aerospace, automotive, and art sectors, binder jetting is utilized for functional testing, fast prototyping, and the formation of complex geometries. It offers great adjustability and a rather quick print speed for large things. It is compatible with a wide range of materials, including metals, ceramics, and sand-based composites (Zhang et al. 2021). Though there are still issues with mechanical strength and surface finish, ongoing study is focused on enhancing the technique and expanding the range of products that can be employed in binder jetting, allowing the technique to be effectively utilized for a broader set of applications (Mariani et al. 2024; Li et al. 2020a; Shanthar et al. 2023).

### Electron beam melting (EBM)

EBM is a modern and efficient additive manufacturing method that uses an electron beam to selectively melt metal powder layer by layer, resulting in complicated and completely dense 3-D structures (Wang et al. 2025). EBM was developed in the 1990 s and operates in a high vacuum environment to prevent the electron beam from reacting with the ambient gases. A small layer of metallic powder is initially placed on the build platform, after which the electron beam is focused across the surface to melt the powder particles in accordance with the digital 3D model (Poomathi et al. 2020). The technique is repeated until the entire shape is produced, lowering the platform when each layer of powder is finished and adding another layer (Konda Gokuldoss et al. 2017). EBM is the preferred material in the aerospace, medical, and defence industries because of its great efficiency and its capacity to manufacture intricate shapes (Ginestra et al. 2020). Vacuum environments reduce contamination and oxidation, producing products with better material qualities (Zhang et al. 2018a). However, to fulfil the potential of this cutting-edge technology, experts are taking steps to address EBM’s constraints, which include a limited material selection, high costs for machinery, and lengthier production periods when compared to other metal 3D printing methods (Dyck et al. 2022).

### Digital light process (DLP)

The invention of DLP technology transformed display and projection technologies. DLP, created by Texas Instruments, reflects light by using a variety of micro mirrors to produce remarkably sharp and clear images. Because of the individual mirror control, it can create high-contrast images with bright colors and deep blacks; this is one of its main features (Traugutt et al. 2020). The response times of DLP projectors are quick. As a result, they work well in presentations, 3D applications, and fast-paced video material (Yao et al. 2021). DLP 3D printing creates sophisticated three-dimensional items by layer by layer using photopolymerization. DLP 3D printers use a light source (typically a projector or an array of UV LEDs) to precisely cure liquid resin, solidifying it into the desired form. Furthermore, compared to their LCD equivalents, DLP systems are typically more energy-efficient and compact (Li et al. 2019). One of the main benefits of DLP 3D printing is its speed; in contrast to conventional extrusion-based techniques, a complete layer may be cured at once, leading to faster print times (Zhao et al. 2019a). DLP is also capable of generating prototypes, jewellery, and extremely detailed models due to its remarkable surface polish and detail capabilities (Zhu et al. 2021).

### Continuous liquid interface production (CLIP)

Carbon3D Inc. invented CLIP in 2015 as a revolutionary and quick 3D printing process (Ligon et al. 2017). By combining the concepts of digital light processing (DLP) with stereolithography (SLA), this ground-breaking method produces objects remarkably quickly and precisely (Janusziewicz et al. 2016). Unlike traditional layer-by-layer approaches, CLIP employs a liquid resin pool and a permeable window to selectively inhibit the process of polymerization on a continuous basis (Johnson et al. 2016). The build platform is raised continually, and the liquid resin is precisely exposed by a high-resolution light projector to solidify it into the required shape (Huang et al. 2021). Faster printing speeds and items with finer surface finishes and isotropic mechanical qualities are produced by the non-stop method (Lipkowitz et al. 2022). Moreover, CLIP eliminates the requirement for support structures; complicated shapes can be printed. This innovative process has paved the way for a new era of additive manufacturing by offering a unique combination of rapidity, precision, and versatility in design. It has been implemented by several industries, particularly consumer goods, healthcare, and automobiles (Deng et al. 2021; Li et al. 2025).

## Effect of 3D printing process parameters

**Fig. 13:**
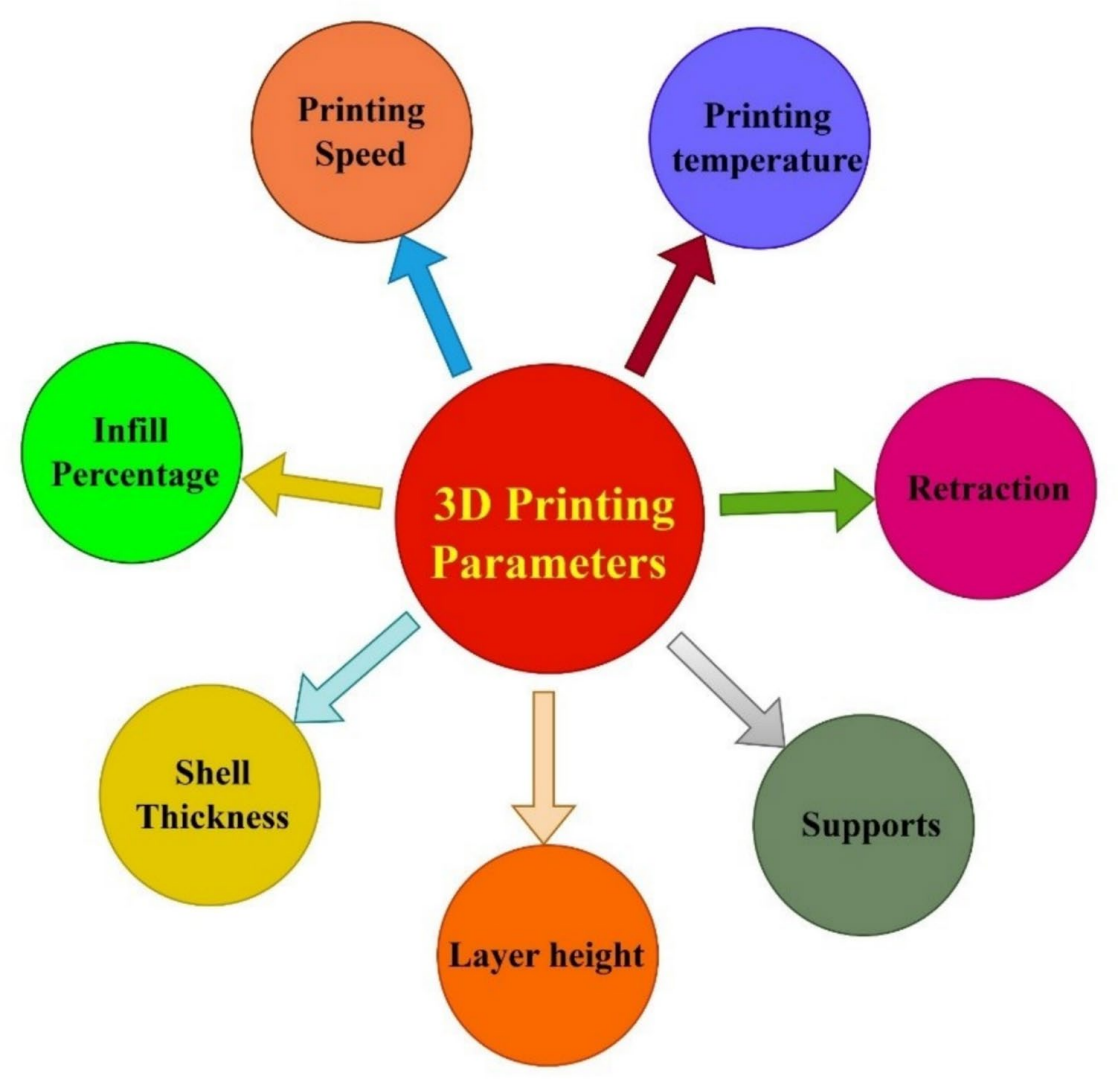
Factors affecting the 3D printing

The response of 3D-printed items is greatly impacted by manufacturing factors such as printing temperature, layer thickness, speed, and fillers density (Qayyum et al. 2022). The necessary mechanical qualities, surface quality, and dimensional accuracy can be obtained by optimizing the parameters that are essential (Singh et al. 2023). Figure 13 depicts the effect of those variables on the overall quality of 3D-printed components, particularly those produced with the FDM technique. The optimization can reduce the time and production cost, besides increasing the robustness and quality of printed parts. A major development has been achieved in the area of process parameter optimization for 3D printing, with technologies such as DOE and machine learning (ML) used to achieve the most effective outcomes. Arrigo et al. carried out a DOE to optimize the extrusion temperature, feed rate, and nozzle diameter for 3D printing in order to produce parts with the best possible quality, developed by using recycled PP (Arrigo et al. 2022). The study discovered that feed rates and extrusion temperature have a major effect on part quality, with nozzle diameter having the second-largest impact. Bakir et al. investigated the implications of process factors on the mechanical properties of recycled PET (Bakır et al. 2021). To achieve the best mechanical efficiency, a number of factors were tuned, including raster angle, nozzle temperature, orientation, infill ratio, and wall structure. The strength and ductility of the printed objects were found to increase with nozzle temperature; however, temperatures beyond 260 °C resulted in filament disintegration. Raster orientation significantly influences mechanical behavior. The parallel orientation will offer the optimum elongation and ultimate tensile strength. The diagonal and perpendicular orientations produced less ductility and ultimate tensile strength (UTS). Horizontal alignment with transverse raster samples has 50% stronger strength and a 100% greater extension at rupture compared to vertical specimens, which failed at a strain of 1%. Moreover, it was discovered that the printed object has to correctly adhere to the bed in order to achieve high yield strength and tight geometric limitations. Also, the best outcomes were attained when the nozzle temperature was between 250 and 260 (°C). The Taguchi method was used by Hartig et al. to evaluate how printing temperature, speed, and nozzle diameter affected the mechanical characteristics of reused PLA. The results of the investigation showed that extruder speed, nozzle diameter, and temperature have a considerable consequence on mechanical performances (Hartig et al. 2021). The impact strength dropped, but the tensile and bending strengths of 3D-printed objects made from virgin and recycled PLA filaments increased, according to a study that examined the relationship between these properties. Reducing the layer height and increasing printing speed increased the tensile and bending strengths (Atakok et al. 2022).

**Fig. 14:**
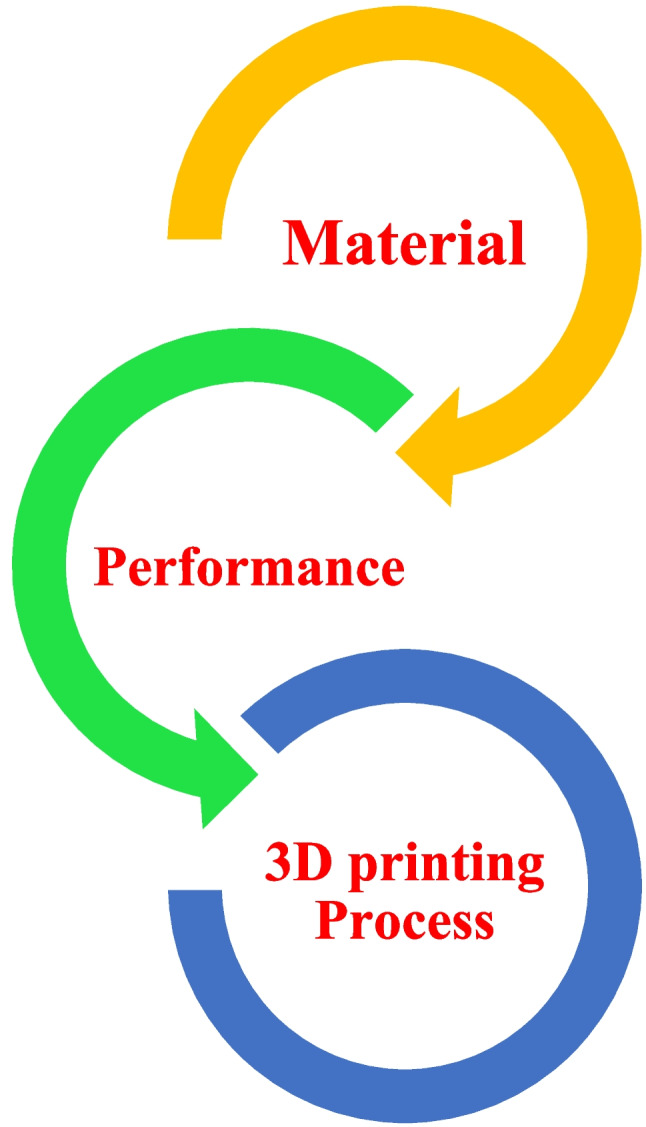
Relationship among 3D printing process, material, and performance

The researcher has conducted a study to assess how printing parameters such as nozzle temperature, printing speed, and layer height affect the mechanical and crystallinity characteristics of recycled PET. The results of the investigation showed that the mechanical and crystallinity characteristics of the recycled PET were significantly influenced by temperature, speed, and layer height. In particular, increased temperature resulted in better mechanical characteristics but decreased crystallinity. Both mechanical properties and crystallinity benefited from faster printing; however, layer height raised had the opposite influence (Van de Voorde et al. 2022). Woern et al. used two reused resources in 3D printing to investigate the effects of nozzle size, layer height, and printing speed on the mechanical properties of PLA and PET. The tensile strength was shown to be highly impacted by both layer height and nozzle size, with larger layer height and nozzle size yielding greater outcomes (Woern et al. 2018b). On the other hand, there was a small impact on printing speed; higher printing speeds led to marginally reduced tensile strength (Woern et al. 2018a). In order to develop a sustainable and biocomposite, Al Zahmi et al. have utilized recycled composite made from different carbon fiber leftover resources to reinforce the increasing waste of PLA as an effective approach to fulfill the demand for the materials. Carbon fiber and their composites were utilized, and different characterizations have explored their potential (Al Zahmi et al. 2022). Horta et al. have varied the base and nozzle temperatures while testing the dimensional exactness of 3D-printed items made of an HDPE/sawdust composite. The results showed that dimensional accuracy was enhanced by raising the base temperature from 60 to 120 °C. A base temperature of 120 °C and a nozzle temperature between 188 and 198 °C were found to be the ideal conditions (Horta et al. 2018). According to Morales et al., the raster angle used in 3D printing has a significant effect on the mechanical properties of recycled PP-based composites with 5 wt.% of cocoa bean shell (CBS). The reason for the higher tensile strength of composites printed at a 0° raster angle as opposed to those printed at a 90° raster angle is the placement of layers along the path of load (Morales et al. 2021b). This observation aligns with the results of a study conducted on recycled PP-rice husk composites by Morales et al. (Morales et al. 2021a). Utilizing recyclable polymers, the investigator has looked at optimizing 3D printing variables to enhance the mechanical properties and overall quality of products. Wall framework, layer height, printing rapidity, nozzle dimensions, raster angle, orientation, infill ratio, and extrusion temperature are some of these parameters. The outcomes demonstrated that these variables have a major impact on the mechanical performance and dimensional accuracy of 3D-printed products. Adjusting these variables can improve the bending strength, ductility, tensile strength, and impact resistance; however, caution must be used to avoid issues like filament disintegration or narrower dimensional limits. The process, material, and performance in 3D printing are interdependent, as shown in Fig. 14. The processes such as FDM, SLS, or SLA indicate the deposition and fusion of the materials, which affects the accuracy, structural integrity, and surface finish. The selection of material determines the printability and properties of the materials. Finally, the performance is the combined outcome of the parameters and material properties.

Artificial intelligence (AI)-based technologies have grown significantly, and they are applicable across various disciplines. AI has proven its importance in the field of material science and has also created dominance with substantial potential that may address the challenges related to energy and environmental concerns by considering sustainability. The exploration of materials through AI is creating a new benchmark in the innovation of novel materials; therefore, AI is serving as a catalyst in the advancement of material innovation. By implementing AI techniques, there is the possibility of creating advanced AI-powered materials. This technique will be helpful in creating a more suitable and sustainable way in the material development for various structural and biomedical applications. The application of machine learning in material science is still at an initial stage, and there are several challenges and difficulties to address in future research. Building a robust ML model requires useful data availability and standardization; therefore, machine learning is highly dependent on data support systems. There is advancement in AI-ML models to build hybrid AI models that create physics-based simulations with ML, which brings high robustness in addressing data scarcity and improving interpretability. Machine learning can make it possible to replace traditional research approaches, even though its potential to create novel materials remains (Bai & Zhang 2025). The preparation of architecture for material selection is a complex process that involves numerous data sets and adjustments across various performances, costs, and performance abilities. In this regard, artificial intelligence and large language models are more preferable for material analysis and information management. This study encourages the use of AI models to reduce the complexity in material analysis and selection; as a result, AI models can potentially reduce the loss of resources and time, and decision-making efficiency can be improved. AI-assisted studies are capable of reducing the time and effort required for material selection (Saad et al. 2024). Nowadays, many logarithmic approaches are innovated for the design and development of novel materials that create huge technological and social advantages. In this context, machine learning is an innovative way that can overcome some of the critical challenges in the path of rational material design and development. Machine learning is transforming the way of thinking to look for complex material designs for novel material findings. It is providing us with a complex ML toolbox that is helpful in the development of data-assisted material design. There may be a possibility that machine learning will be integrated into all components of material design. The interesting fact about machine learning models is that once a discovery or prediction is formulated, the paths of decision-making can be traced back to uncover new insights (Moosavi et al. 2020). It is demonstrated that AI and ML are rapidly gaining traction in material innovation and its design. In addition to the typical approach of fitting a model to a huge amount of data and making predictions, the material community is developing innovative and relevant ways to incorporate AI into their work. This has accelerated not only material design and discovery but also other areas of material research. It is also evident that we require AI/ML approaches and models that are better suited to material research (Reyes & Maruyama 2019).

## Challenges in manufacturing of biocomposites by AM

The mechanical performance of 3D-printed objects has been enhanced by the advancement of fiber-incorporated polymer matrix filaments (Nagarjun et al. 2023). Nevertheless, further comprehensive investigation remains required to configure the 3D-printed structure and satisfy the structural application's specifications. Manufactured composite structures are mostly plagued by inherent extrusion-induced faults such as voids or porosity that arise from insufficient bonding between the inter-printed layers and between the fibers and the matrix arrangement, in addition to low surface finish. Distinguishing between the filament’s chemical and physical properties and its printed structure is crucial. Moreover, incorporated reinforcements affect composite materials’ printability due to their structure and geometry (Wang et al. 2017). Despite the availability of several research articles outlining the benefits and drawbacks of 3D-printed composites made of synthetic materials, there is a dearth of investigation on the performance of biocomposites. To choose biofibers’ suitability, it is essential to know their individual characteristics, including their microstructure and constituent qualities. To achieve optimal performance, it is critical to take into account their inherent defects, natural changeability, and reaction to various environmental service conditions such as heat and humidity (Baley et al. 2019).

**Fig. 15:**
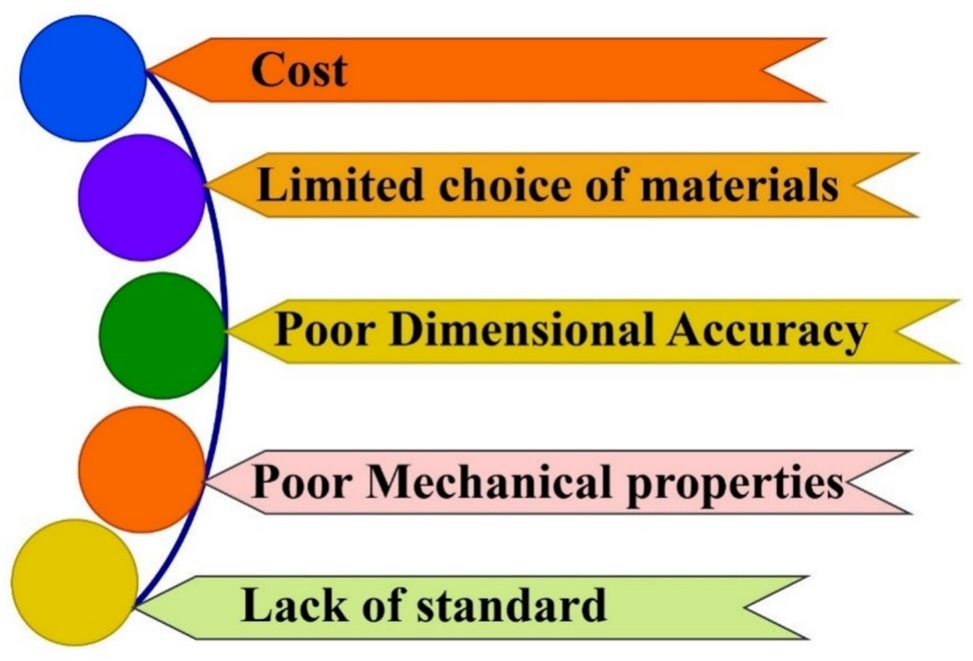
Challenges in 3D printing technique

Due to the several barriers associated with 3D printing as given in Fig. 15, 3D printing is not widely used. The use of 3D printing for a wide range of industrial objects is being restricted by the expensive price of ingredients, the starting price of the technology, and the shortage of skilled labor (Go et al. 2017). In addition, a number of challenges, including low dimensional precision and poor reproducibility of material properties, make the process unreliable in the eyes of material researchers and manufacturers (Fu et al. 2023). The insufficient mechanical qualities of the final product, combined with poor surface polish, limit the usage of 3D-printed parts in a variety of crucial applications (Yadav et al. 2024). Notwithstanding, these problems can be addressed by examining the impact of diverse process variables on mechanical attributes and generating supplementary understanding of the procedure, thereby enhancing the processes’ efficiency in producing superior items.

Another concern with the process of additive manufacturing is printing duration. AM techniques are frequently laborious and slow. A reduction in printing time is necessary for AM to take the role of traditional manufacturing. Although AM systems are expensive, this might not be a problem in a few years. Although there are currently some affordable, customizable small 3D printers on the marketplace, massive production setups are expensive. Not only is it an expensive tool but it is also facing necessary supplies including filaments. Depending on its specs, a 3D printer filament can cost anywhere from $25 to $50 per unit (Kim 2018). Anisotropy of additively built structures is one of the most significant issues in the current context. This might cause mechanical qualities to fluctuate depending on the loading conditions. 3D-printed objects have an unsatisfactory aesthetic when viewed from the side; they may display the layer-by-layer printing sequence (Pérez et al. 2020). This flaw could be insignificant for scaffolding, but it matters a lot for structures and toys, where aesthetics are important.

## Effect of ambient conditions on the performance of biocomposites

The surrounding environment has a big impact on the biomaterials’ characteristics. The various elements that affected the properties of biocomposites were covered in this section. Additionally, the impact of expedited weathering on biocomposites is examined.

### Moisture absorption behavior of biocomposites

The occurrence of an advanced fraction of hydroxyl groups (OH) favors the hydrophilic performance of the biocomposites. However, not all the elements present in the biocomposites induce water absorption. Water molecules can enter the amorphous part of cellulose in biofibers because there is a small portion of cellulose that is semi-crystalline in nature. On the other hand, hemicellulose is much more amorphous, which makes it more susceptible to moisture intrusion. Nevertheless, lignin is hydrophobic (crystalline). Due to the diffusion of moisture into the micro-capillary or gap between the micro-fibrils, a biofiber swells up when it is exposed to hydrolytic aging. The presence of moisture in the fibers can create a monolayer or a multilayer that can make partial or complete contact with the OH group (Nurazzi et al., 2021). Moisture absorption in biofiber-reinforced composites depends on a number of variables, including the fiber volume fraction, the orientation of the reinforcement, temperature, humidity, and diffusivity. The moisture in biocomposites is governed by three mechanisms. The process starts with capillarization, after which water molecules infiltrate the matrix networks by micro-damage at the fiber/matrix contact. Furthermore, it is possible to categorize moisture diffusion of a material from highest to lowest concentrations (Zhang et al. 2014). The most prevalent process is the capillarity of water fragments through micro-cracks and holes.

**Fig. 16:**
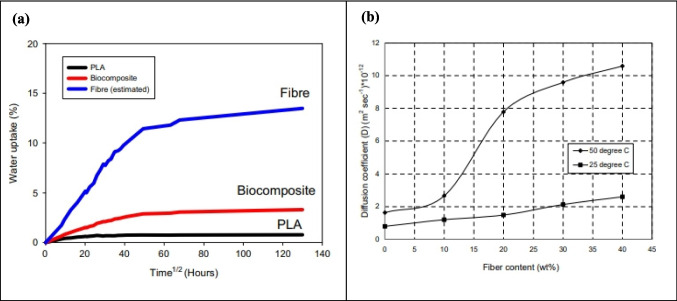
**a** Water uptake behavior of pure PLA, biocomposite, and fiber (Le Duigou et al. 2014). **b** Diffusion coefficient for composites at various temperatures (Osman et al. 2011)

Water molecules moving through the matrix and the interfacial region can potentially result in tiny fractures. Biofiber-based composites expand as they absorb moisture because of hydrolytic aging because biofibers are polar. This leads to the formation of internal tension at the fiber/matrix interface, the expansion of the amorphous fiber segment—a phenomenon called dilapidated—and small cracks in the polymer enveloping the reinforcements. Water molecules absorbed into the material cause swelling, fiber/matrix debonding, plasticization, and hydrolysis. The fiber-matrix barrier is weakened, and increased moisture absorption is caused by this micro-damage. The poor mechanical properties result from the reduced load transfer efficiency of the system caused by the inadequate fiber/matrix interaction, as shown in Fig. 16a and b. The cyclic absorption and desorption of water molecules over a broad humidity and temperature range are common causes of fatigue degradation in biofiber-based composites. Furthermore, the examination of the water absorption reaction is important because of fatigue deterioration in the inter- and intra-laminar locations of the composites, which may be caused by recurrent deviations in the internal stresses due to moisture absorption. These effects can affect the mechanical properties of a material and its long-term longevity.

**Fig. 17:**
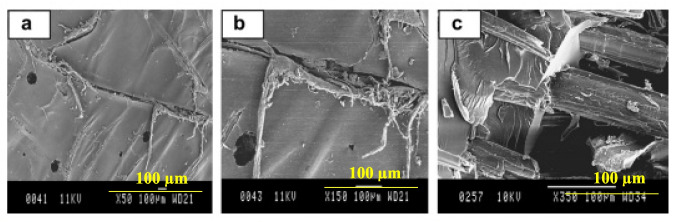
Failure showing **a** matrix cracking, **b** fracture running along the interface, and **c** fiber–matrix debonding due to attack by water molecules (Dhakal et al. 2007)

Zaki Abdullah et al. examined how moisture, water spray, and UV radiation have affected the tensile and flexural behavior of kenaf-PET/polyoxymethylene (POM) composites throughout atmospheric aging. The samples require roughly 672 h aging in an accelerated environmental unit. Tensile stress reduces by 50% and 2% for the kenaf/POM and hybrid kenaf-PET/POM composites, respectively. This finding indicates that the hybrid composite outperforms the uniform composites in terms of water resistance (Zaki Abdullah et al. 2013). In contrast to the hybrid kenaf-PET/POM composites, the homogeneous aging shows a significant decrease in the tensile characteristics of the kenaf fiber due to the degradation of the cellulose, hemicellulose, and lignin content. The researchers have worked to find out how the absorption of moisture affected the mechanical properties of hemp/polyester composites that were hydrolytically aged for varying amounts of time at 25 °C and 100 °C (Dhakal et al. 2007). When samples are exposed to water at higher temperatures, they absorb more moisture than when at normal temperatures. This was explained by the fact that the composites exposed to higher temperatures produced more micro-damage as given in Fig. 17a–c. According to Chen et al., the development of more hydrogen bonds between the cellulose and matrix causes the interfacial shear strength of the bamboo-based biocomposite to drastically drop as humidity levels rise (Chen et al. 2009).

### Effect of temperature on behavior of biocomposites

Temperature is a significant influence on the deterioration of biocomposites under environmental service conditions. The influence of ambient temperature on the mechanical characteristics of biocomposites is not fully explored. According to Kumar et al., damage caused by impact stresses on hemp, basalt, and epoxy has negative effects as the temperature rises. The hemp/epoxy and hemp/basalt/epoxy hybrid composites outperformed basalt/epoxy composites in terms of durability against impact at 50 °C (Kumar et al. 2015). Mueller has demonstrated that all composite materials behaved similarly, through the maximum impact resistance in the medium temperature range regardless of the type of reinforcement. Additionally, there is a somewhat typical weakening at both lower and higher exposure temperatures (Mueller 2004). According to Shen et al., slightly higher temperatures may be able to repair the harm that impact loads have caused to composites made of flax fiber (Shen et al. 2019). The influence of temperature on the impact behavior of jute/unsaturated polyester composites was studied (Hachemane et al. 2013). It was observed that at an ambient temperature, the composite could withstand greater stresses. According to David-West et al., biofiber/styrene polyester composites exhibit some degree of flexibility in the post-impact phase at elevated temperatures (David-West et al. 2011). The damages caused by the reduction of the toughness and modulus of the composite could be the possible cause of the abrupt change in the load of flax fiber at 40 °C and 60 °C.

### Effects of Cryogenic temperature on the performance of biocomposites

An understanding of the effect of extremely low temperatures on the mechanical properties of biocomposites is important for verifying the suitability of biocomposites in structural applications. The performance of biocomposites can be greatly impacted by the cryogenic temperature, which is thought to be exceptionally low and typically falls below −150°C (Lavorel et al. 2021). Cryogenic experiments for glass and carbon fibers have been demonstrated in certain works. However, there is limited work available on the performance of biocomposites in cold environments (Nassiopoulos and Njuguna 2015; Velmurugan et al. 2023). There are several varieties of cryogenic liquids accessible, including liquid oxygen, hydrogen, nitrogen, and helium. Typically, specimens are submerged in these liquids at various intervals and evaluated at temperatures below −150°C.

### Effects of ultraviolet on the performance of biocomposites

The electromagnetic radiation produced by the sun is the source of UV rays. The surface oxidation and color change brought on by the photo-degradation reaction are the direct results of UV exposure. This phenomenon results in physical changes such as color fading, deterioration, surface defects, and microcracking in addition to the dissociation of chemical connections between the polymers. The UV degradation may accelerate much more at higher temperatures. It is important to understand that under certain environmental exposures, the influence on characteristics will also be negatively impacted by increases in exposure time, temperature, and relative humidity. Exposure to ultraviolet radiation can weaken the bond between a fiber and the matrix, especially at higher temperatures and for longer periods of time. When it comes to outdoor applications, biofiber-reinforced composites are always vulnerable to UV radiation, which can significantly impair mechanical efficiency, overall strength, and lifespan. Yan et al. examined the surface glossiness of composites at various exposure times such as 0, 500, 1000, and 1500 h. It is observed that as the exposure time extended from 0 to 1500 h, the composites experienced significant color fading (Yan et al. 2015). It was hypothesized that the composite’s diminished shine was caused by the deterioration of the flax fiber and epoxy matrix due to exposure to sunlight over time. Surface oxidation brought on by UV radiation increases the surface and interior mechanical and thermal stresses in composite materials. These occurrences have an impact on the composite surface and ultimately cause polymer chain breaking, which concentrates stress and causes shrinkage, lowering the overall performance of composites (Olivier et al. 2009). To comprehend how UV light exposure affects different properties and damages composites, it is essential to investigate the effects of this exposure on these qualities. Biocomposites are extremely vulnerable to prolonged and high temperatures when exposed to UV radiation.

### Effects of accelerated weathering conditions on biocomposites

The combined effect of accelerated deterioration, especially UV radiation, and being exposed to harsh conditions such as high temperature, humidity, and wetness has influenced the composites’ durability over time, structure, and degrading tendency. The various composite materials perform variably in different accelerated weather situations. Biofiber-reinforced composites, due to their chemical composition, can become fragile at high temperatures. Hemicellulose degrades at temperatures above 160 °C. Thus, it is vital to understand how this exposure impacts biocomposites’ properties and damage trends. According to Dayo et al., the characteristics and color of the biocomposites are greatly impacted by exposure to different accelerated weathering environments (Dayo et al. 2020). It was found that when hemp composites were exposed to accelerated weathering conditions, their flexural characteristics decreased. Moreover, it was demonstrated that the plasticizer effects resulting from the hydroxyl group (-OH) production in hemp fibers raised the strain values for samples that were subjected. Mechanical effectiveness is expected to be significantly reduced as a result of the exposed fiber-matrix interfaces of composites weakening due to enhanced weathering conditions. An examination of the impact of rapid UV weathering on the properties of flax epoxy composites was presented (Yan et al. 2015). It was revealed that when compared to non-exposed samples, accelerated weathering causes a reduction in tensile and flexural properties. This loss of mechanical characteristics can be caused by weathering-induced degradation of the fiber-matrix interfacial connection.

### Hostile solutions

Despite the fact that a large number of studies have been conducted on the effects of hostile environments (acid and alkaline) on synthetic composites (Kevlar/epoxy and carbon/epoxy laminate (Askari et al. 2023), carbon/cork filled epoxy laminates (Reis et al. 2020), and Kevlar/epoxy composites (Reis et al. 2021) and limited research has been conducted on natural fiber composites (Sgriccia et al. 2008; Bledzki et al. 1996). Nonetheless, natural fiber composites can also benefit from the same principles. The mechanical characteristics of the materials are considerably impacted by the aggressive solutions; however, the effects of these solutions are highly concentration-dependent. In these extreme settings, they would have considerably more damaging impacts on biobased natural fiber composites. It is crucial to look into how they affect the biocomposite.

## Applications of biocomposites

**Fig. 18:**
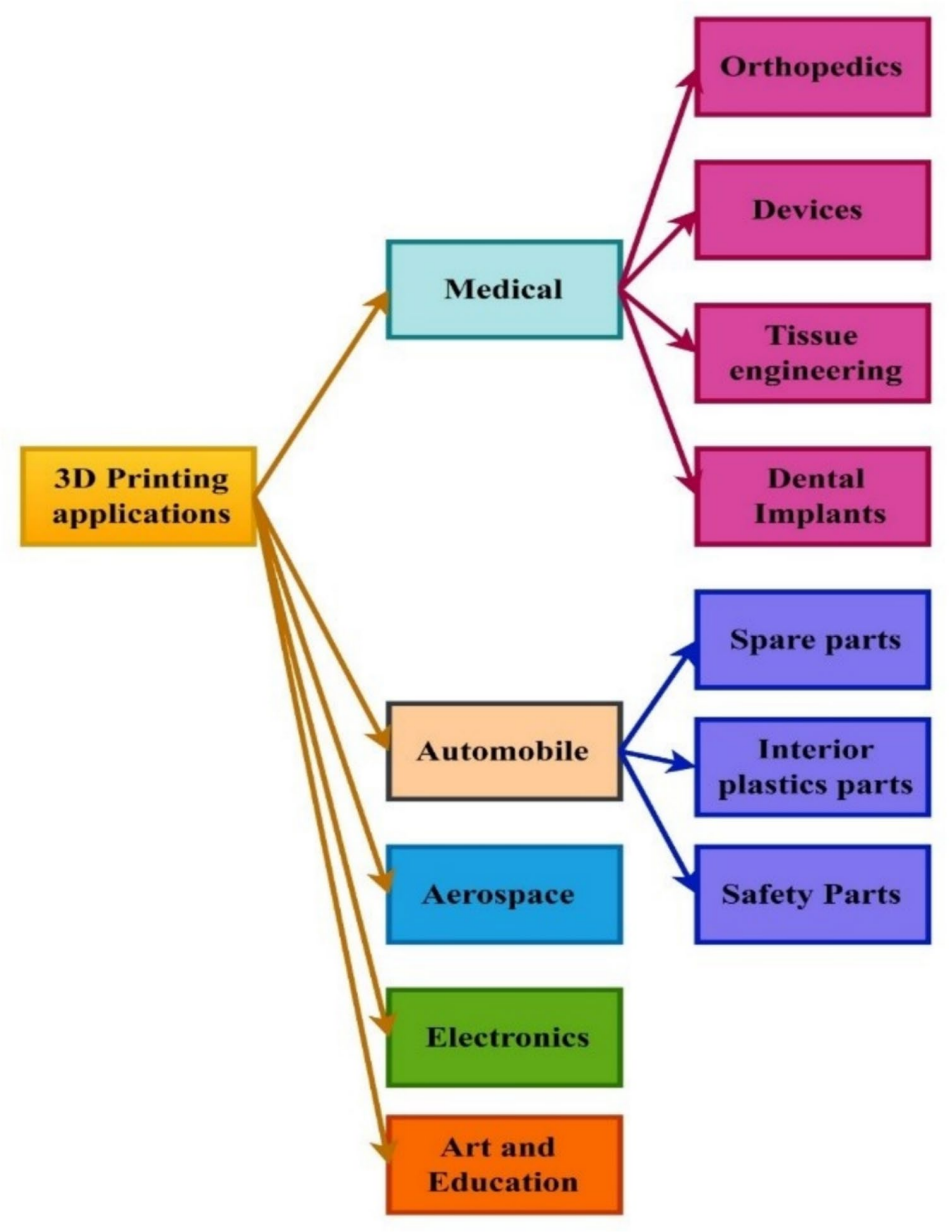
Applications of 3D printing parts

Biocomposites are currently gaining popularity as a potential substitute for metal, conventional reinforcement-based composites, and ceramic-based materials in healthcare applications, aerospace, automotive, marine, sports, packing, and electronics sectors, among others. Moreover, biocomposites are used in electromagnetic shielding, coating, packaging, electrical, medicinal, magnetic, and optical applications, as shown in Fig. 18 (Sarasini and Fiore, 2018). 3D printing offers an opportunity to transform medical research by allowing the creation of complex medical equipment like implants, exoskeleton pieces, orthopedic and dental materials, and tissue engineering (Buchanan and Gardner 2019) and anatomical models (Lozano et al. 2018). The ability to customize products with 3D-printed parts expands the market for these parts to include the fashion industry. When compared to traditional glass fiber-reinforced composites, biocomposites are noticeably lighter. As a result, biocomposites have been widely used in automobiles to enhance fuel efficiency and reduce emissions of carbon dioxide. Due to their poor mechanical performance and natural moisture resistance, biocomposites are commonly employed in interior components such as door panels, matting, storage compartments, dashboards, and so on (Li et al. 2020b). Biocomposites utilized for external components include abaca-based biocomposites for wheel covers, spoilers, fender sections, cabin floors, and flax/polyester biocomposites in engine and gearbox casings (Muhammad et al. 2021). Biocomposite boards are used in railcars in India. Biofibers absorb moisture and maintain humidity, which contributes to improved interior comfort in cars. Biocomposites are also used in the construction of turbine blades (flax/polyester) and interior panels for aircraft. Biocomposites based on jute fibers are now used in the housing industry for interior and structural applications such as wall insulation, bathtubs, roofing panels, hurricane-resistant housing, door and window frames, and floor lamination. Biofibers are suited for tooling equipment because of their non-abrasive quality. Additionally, biocomposites are used in masonry, furniture, helmets, protective covers, mirror casings, roofing, voltage stabilizers, clothing, recreational equipment, fishing rods, outdoor gear, archery bows, string instrument top plates, and green materials in analytical chemistry (Pickering et al. 2016). Surfboards or eco boards for commercials are made by using hemp fiber and biobased polymer. The marine sector is another significant industry area that has studied sustainable lighter biocomposite materials. Glass fiber-reinforced polyester composites are currently mostly utilized in parts linked to marine engineering. However, eco-friendly biocomposites are being looked for as a substitute material because of their challenges with recycling. Nevertheless, their complete uses in this industry are limited due to the inherent moisture problems. Some positive steps are being taken to lower these obstacles.

AM has been growing its reach in practically every sector. In the automotive sector, the global market share of AM is $1.61 billion in 2020 and is predicted to increase to $4.89 billion in 2027 (Malik et al. 2022). AM is an effective technique for the manufacturing of composites for complex automobile parts since it optimizes materials and reduces waste. 3D-printed composites can be used in lightweight and high-strength components such as brackets and structural elements of defence and aerospace. AM has been widely employed in the medical field as the techniques allow for the development of 3D-printed composites for implants, prosthetics, surgical templates, and dental restorations with high precision. Its market share is expected to grow from $89.3 million (in 2019) to $348.1 million by 2027 (Rouf et al. 2022). AM is used in a variety of medical fields, including cardiothoracic surgery, neurology (Randazzo et al. 2016), orthopedics, and plastic surgery (Chae et al. 2015). A recent area of AM is bioprinting which considers the production of skin and other tissues. AM has grown tremendously in prosthodontics allowing dentists to scan, prepare, and print teeth. AM has been used to print crowns and dentures, both removable partial and complete (Tian et al. 2021). The use of AM has resulted in significant advances in maxillofacial surgery. The most important use of AM in orthopedics is the creation of scaffolds with specific porosity, pore shape, and size. 3D-printed composites can be used in building construction such as building blocks, concrete structures, and models for architectural designs, in housing materials, and street furniture. The industrial composites such as design elements, furniture, and machinery components are developed by using 3D printing.

## Circular economy in biocomposite

**Fig. 19:**
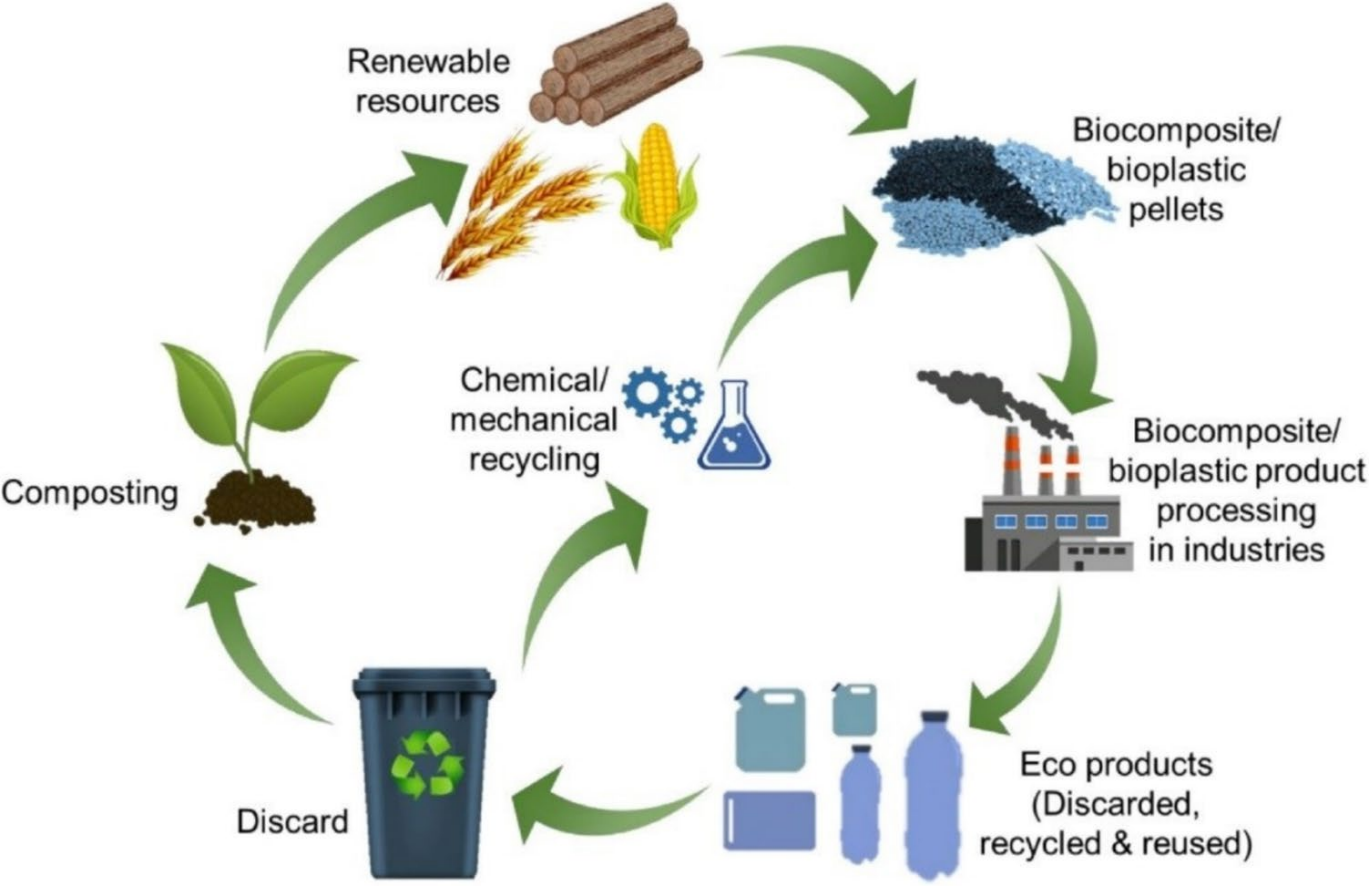
Recyclability of biocomposites (Shanmugam et al. 2021)

The issues associated with the mismanagement of plastic wastes have triggered the world to develop effective plastics waste management techniques. As such, numerous initiatives have been undertaken to limit plastic usage and investigate incineration and recycling of plastics as potential approaches to reducing the harmful effects of plastic trash (Chen and Yan 2020). However, the burning of polymers deteriorates the air and water due to the detrimental effects of chemical and ash waste residue released into the environment (Webb et al. 2012; Yang et al. 2021; Qureshi et al. 2020). The incineration of plastic wastes requires energy, which is supplemented by non-renewable petroleum-based resources such as coal, liquid fuels, etc. Despite the high operation cost of incineration of about 5.53 USD billion per year, the process fails to diminish the problem of plastic wastes. The challenges associated with recycling plastics include sorting, collection, lack of transport and handling facilities, unskilled workers, etc. (Hopewell et al. 2009). Therefore, the usage of bioplastics has been identified as an effective approach to shrink the harmful results of plastic wastes. The composites based on bioplastic can effectively replace plastic-based composites. The biodegradable reinforcement, such as agricultural wastes, can be utilized in the development of biocomposites (Gurunathan et al. 2015). The biocomposites are sustainable and can be a suitable alternative to the conventional composites. Furthermore, the creation of bioplastic-based composites permits the efficient use of biomass waste. The market for biocomposites is expected to rise at a compound annual growth rate of 11.2% between 2017 and 2030 (Moshood et al. 2022). The reprocessing of biobased composites can effectively fulfill this requirement. The recycling of biocomposites is denoted in Fig. 19.

**Fig. 20:**
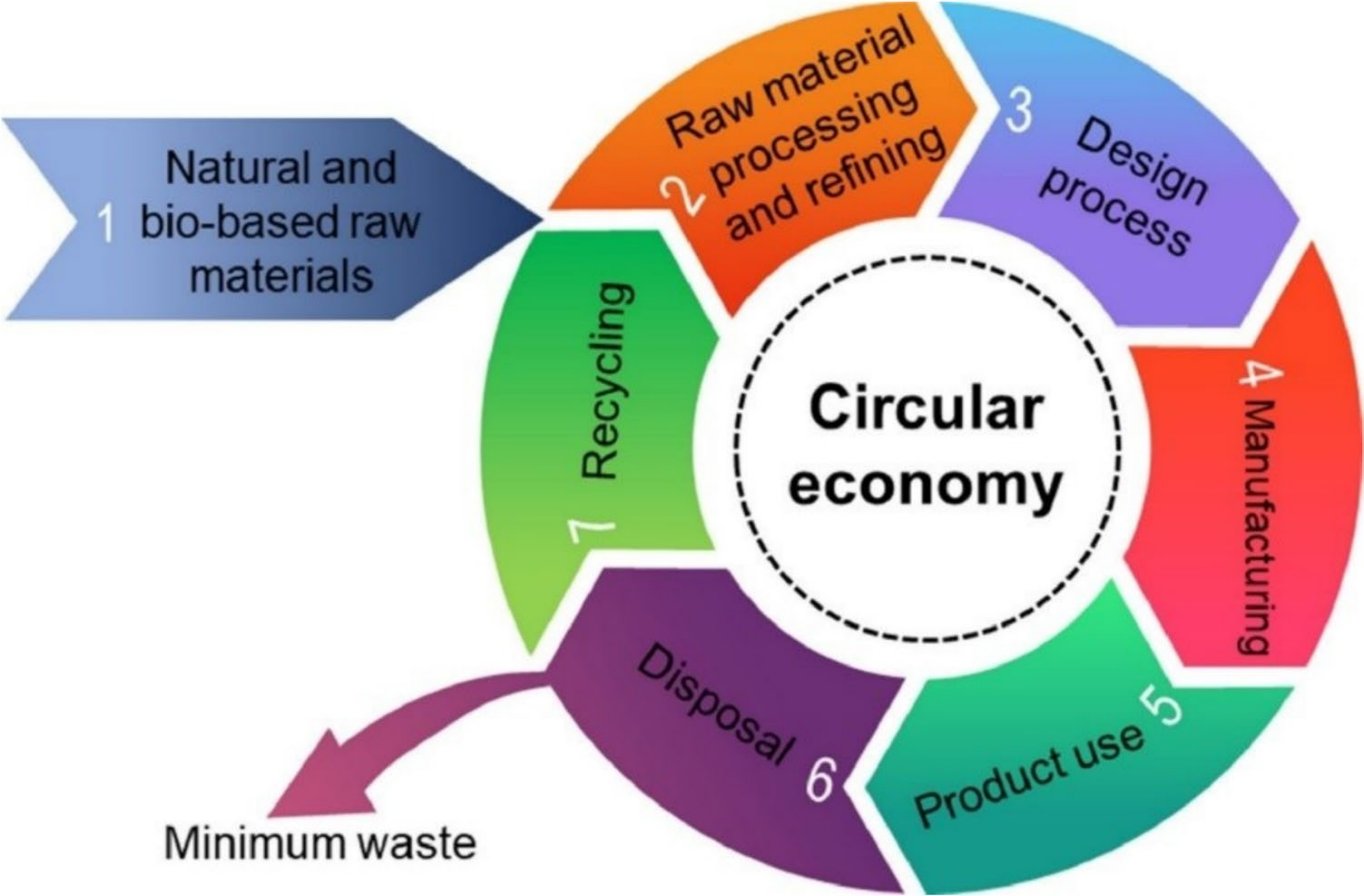
The different phases of the CE model (Shanmugam et al. 2021)

The growing need for the use of natural resources in biocomposites manufacturing has highlighted the importance of a circular economy (CE) in biocomposites, enabling recycling and reuse (Lamberti et al. 2020). The goal of CE in biocomposites is to preserve sustainability by reducing waste and maintaining the resource at optimal levels. Although the reduction of carbon footprint at every stage of the product life cycle is a concern of CE, the utilization of waste for the development of composites is one of the ways to implement CE (Winans et al. 2017). The implementation of CE in biocomposites increases reusability, recyclability, and transforms biocomposite waste into useful energy, products, or secondary material, which reduces the waste produced in biocomposites as landfilling (Loiseau et al. 2016). The conversion of waste into value-added products can decrease landfilling and ensure the uninterrupted utilization of raw materials in CE, thereby allowing the conservation of valuable resources (Ayre 2018). The inherent bonding of the polymers and reinforcement increases segregation costs, thereby challenging the implementation of CE. The adoption of circular practices in biocomposites, such as reuse, recycle, repair, and design, can assist in the implementation of circularity in biocomposites (Reike et al. 2018). The Institute for Advanced Composites Manufacturing Innovation (IACMI) has promoted a circular economy in composites by reusing and recycling fibers. It has been established that the recovered fiber from composites uses 15% less energy in compressing than that required for the manufacturing of virgin carbon fibers (Shanmugam et al. 2021). Figure 20 depicts the different phases of the CE model for composites. The utilization of materials made from biomass, recycling, and Life Cycle Assessment (LCA) are the three key elements influencing CE implementation in biocomposites.

### Benefits of circular economy in biocomposites

**Fig. 21:**
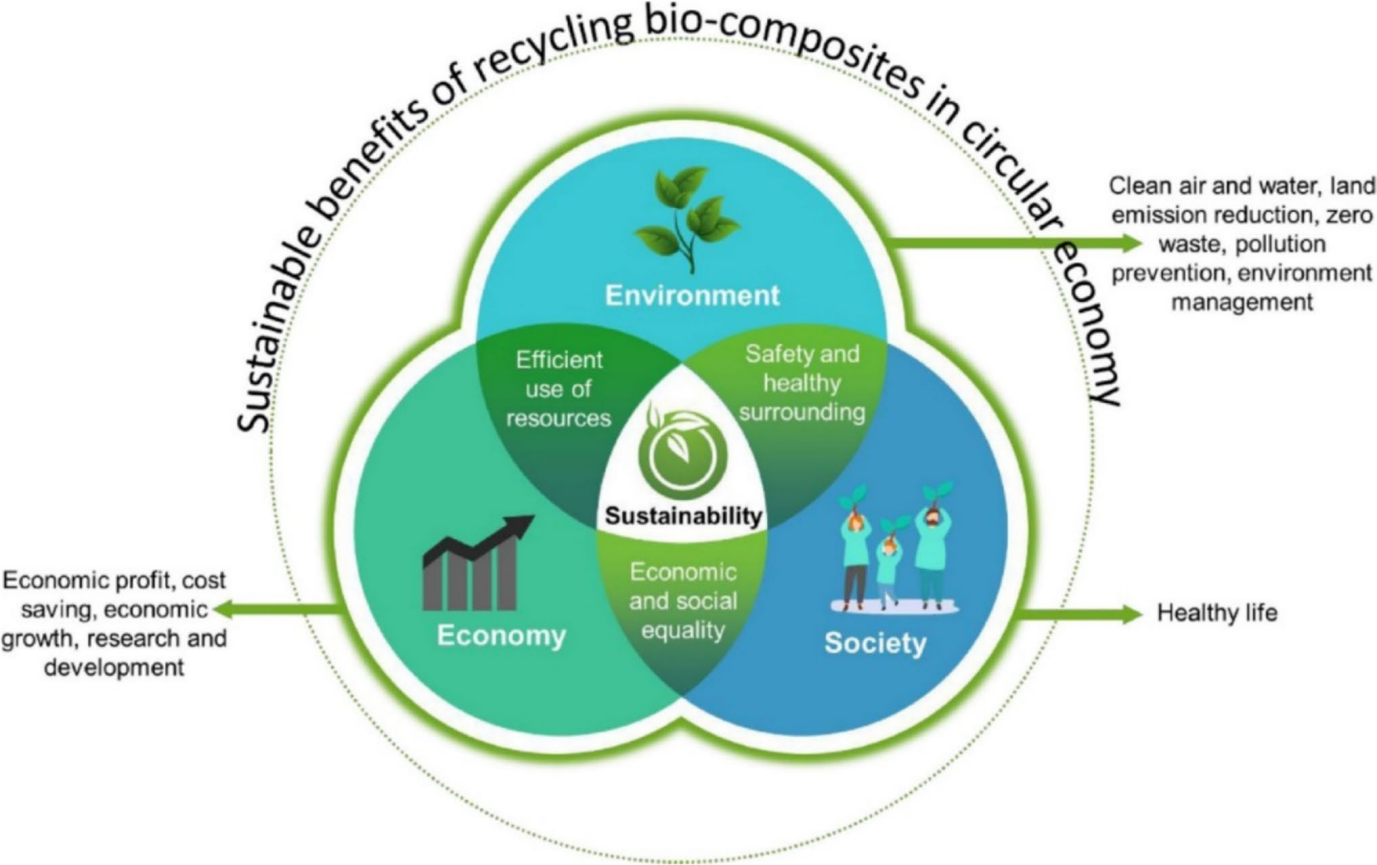
Sustainable benefits of recycling biocomposites in CE (Shanmugam et al. 2021)

CE in biocomposite will bring socio-eco and environmental benefits, as given in Fig. 21. A huge amount of energy, resources, and cost is acquired in the development of composites. The disposal of composite in landfills has resulted in a huge loss of materials and economy, eliminating the possibility of reusing these valuable materials. Therefore, there is a need for the growth of business strategies for the recycling of these wastes through the implementation of a circular economy model (Mativenga et al. 2017). The process used for improving composites’ recyclability is significant for both the user and the manufacturer’s financial advantages. The recovery of materials, which saves energy usage and economic costs while also lowering environmental effects, are the benefits of recycling. These causes are driving interest in the recycling of biocomposite. The recovered matrix and reinforcement can be used again by mixing them together or separately for different applications. Therefore, the adoption of a reliable recycling process for the recovery of material from scrap is the goal of the current research in industries. An effective recycling of biocomposites improves productivity and sustainability in CE. Also, the CE approach for biobased composites provides motivation for successful recycling and reuse of biodegradable polymers and reinforcements. The CE approach makes for efficient and effective end-of-life treatment of the polymeric waste, which is a serious threat to the environment, thereby highlighting the significance of biocomposites. The researcher has worked on the sustainable design of the composites and has explored the prospects of CE (Sauerwein et al. 2019). The recovery of materials to ensure the stability of materials and the material life cycle are the major components of the design of biocomposites concerning CE (Den Hollander et al. 2017).

#### Economic impact of biocomposites

Biocomposites exhibit a lesser environmental effect as compared with traditional composites. However, the commercial applicability of a sustainable product is heavily influenced by its cost (Roy et al. 2019). The biocomposites developed by using natural fiber have successfully decreased the cost of the biocomposites. The economic impact of the biocomposites developed by using banana fiber has been studied by applying a life cycle cost performance method where the production cost of the composites was grouped into machining cost, material, energy, and labor. It was observed that despite the low cost of the banana fiber, the material cost was increased due to the pre-treatment of the banana fibers required to increase the binding of the fiber and matrix (Vinayagamoorthy 2021). However, the banana fiber composite has a 17% lower cost of production than the polyester resin. Furthermore, replacing glass fiber with natural fiber reduces the production cost of biocomposites by 80% while decreasing composite costs by 5% (Venkateshwaran et al. 2011). According to worldwide market studies, the worldwide use of biocomposites will be 19.6 billion dollars in 2020 and 38.07 billion dollars by 2025 with a compounded annual growth rate (CAGR) of 14.2%. In contrast, the global market for synthetic fiber was 147.16 billion dollars in 2019 and is expected to grow at a 5% CAGR by 2025, indicating a decline in utilization rate (Gurunathan et al. 2015). Furthermore, plant fiber output has grown at an exponential rate throughout the last 20 years, with projections of 145 million tons by 2023, which has increased from 107 million tons in 2018 (Junghare et al. 2023). The technological innovations have necessitated the search for novel composite materials and applications. The investigations on the cost analysis for the commercialisation will increase the economic viability of the biocomposites.

#### Environmental benefits of biocomposites

Biocomposites are made from natural reinforcements and polymers that decompose quickly, and they may be simply recycled or destroyed without leaving any detrimental product, making them ideal for the circular economy. PLA’s organic nature makes it easily decomposable by microorganisms (Song et al. 2009). Moreover, burning or landfilling PLA produces a lesser amount of toxic substances and leachates as compared to other plastics. It is reported that around 30–50% less fossil fuel is consumed by PLA, which decreases the emission of CO_2_ by 50–70% when compared to oil-based plastics (Vink et al. 2003). The study has reported lower emissions of CO_2_ for the PLA material as compared to PET. The type of blending material, reinforcement, and systems boundaries all have an impact on PLA's greenhouse gas (GHG) emissions. The blending of bioplastics with synthetic plastics can improve the properties of bioplastics and allow for modifications in properties according to the desired applications (Madival et al. 2009).

Thermoplastic starch (TPS) is another bioplastic that is rigid, biodegradable, and biocompatible (Chong et al. 2021). TPS possesses good biodegradability in soil and water; however, it suffers due to poor mechanical strength and hydrophobicity. The low moisture content and short life span are the limitations of TPS, while it can be successfully blended with other plastics. The work has demonstrated the development of wood-reinforced composites blended with different fractions of PLA and TPS, i.e., 30% wood fiber + 35% PLA + 35% TPS (Mohammadi Nafchi et al. 2013). Life Cycle Assessment (LCA) of the wood/PLA composite has found a better response for global warming and ozone depletion. The comparative analysis for the ozone depletion of TPS and Wood/PLA composites has observed a reduction in ozone depletion with the blending of TPS. The upstream manufacture of PLA demands additional input power, which contributes to decreasing ozone levels for PLA composites. TPS has contributed considerably, about 18.05%, to the reduction of ozone depletion, while a little contribution of about 1% is observed for the wood. The study has revealed that blending TPS with other plastics improves environmental performance (Mahalle et al. 2014). A different investigation assessed the environmental performance of the PLA-blended starch material and compared the results to PS. According to the research, PLA/starch material influences the global warming potential (GWP) factor by accounting for 73–97% of the entire actual score (Peantham et al.,^ 2024^).

PHA are recyclable and biodegradable bioplastics which consist of polyesters of R-hydroxyalkanoates acid (Basnett et al. 2016). The researcher has blended PHA in wood composites for the development of two different types of composites, namely, PLA wood composites comprising 20 wt. % wood fiber + 80 wt.% PLA, and PHA mixed wood composites composed of 20 wt.% wood fiber + 55 wt.% PLA + 25 wt.% PHA. The CO_2_ emissions for the PHA blended composites were 3591 kg CO_2_ eq./*t*, although the PLA composite gives a CO_2_ emission of 3742 kg CO_2_ eq./*t*. The study has suggested that the integration of PHA improves the sustainability of PLA composites, but the study has used the cradle-to-gate method for LCA valuation; as a result, it fails to demonstrate the utilization of product and end-of-life phases. The reinforcement of natural biomass in bioplastics can successfully reduce the cost of bioplastics. Moreover, the strength of the biocomposite increases with the reinforcement of natural fiber while reducing carbon footprints and emissions of GHG. The carbon footprint of natural fiber was 80% lower than that of synthetic and glass fiber (Qiang et al. 2014). The carbon footprints for the production of each tonne of glass fiber are 1.7–2.5 tonnes of CO_2_–eq and 0.3–0.5 tonnes of CO_2_–eq for natural fiber. The work has explored the methods that can be used to integrate circularity and the principles of a CE into the production systems of plastics. It is established that while CE aims to eliminate waste through design, it must be advanced by taking into account the impact of raw materials, the complete product value chain, and end-of-life alternatives to achieve sustainability. The discussion highlights the growing field of biodegradable, low-carbon polymers derived from renewable sources, focusing on their technical and environmental benefits that aid in lowering carbon footprints. The research conducted from a sustainability perspective, which assesses CO_2_ emissions from the production phase to the end-of-life stage, is examined (Amulya et al. 2021).

The evaluations of the GHG for the sisal fiber and glass fiber have found a lower GHG emissions of 70–90% for sisal fiber when compared to the glass fiber (Broeren et al. 2017). The results have suggested that the reinforcement of natural fiber in bioplastics for lowering the GHG houses and promotion of sustainability. Biowastes have been used as filler in the production of environmentally acceptable biocomposites. Agricultural wastes such as rice husk ash and wood floors were used to create polybutylene composites (Kim et al. 2005). The soil burial test was conducted to explore the biodegradability of the biocomposites. A linear relationship for the biodegradability was observed from the date of burial; also, the composites lose weight by 5 to 10% over 120 days of burial. Ambone et al. have developed the PLA-based biocomposites by utilizing waste leather buff as reinforcement and found an enhancement in the tensile strength with the reinforcement of 10 wt. % of waste leather buff as compared to the neat PLA (Ambone et al. 2017). The study has demonstrated the potential of waste leather buff and reduces incineration of leather waste (Das et al. 2016). Recently, biochar produced by the pyrolysis of biomass has gained considerable interest as filler in the development of composites. The transformation of biomass into biochar reduces the emission of GHG as it prevents the decay of biomass (James et al. 2022). The work has demonstrated the environmental impact of PP and biochar composites by considering two different types developed by reinforcing 30 wt.% of biochar and 20 wt.% of talc. The biochar composite has shown a lower environmental impact of about 25% as compared to the talc-reinforced composite (Tadele et al. 2020). The loading fraction of filler and matrix significantly influences the environmental impact of the composites, and further optimization in the loading ratio will improve the environmental impact of the composites.

## Future trends biocomposites through AM

AM is a potential technique for producing short or continuous biofiber-based composites. Future attempts to produce high-quality biocomposites via AM may involve multiple pathways. The optimization of the filament feedstock with low twisted fibers at the material level allows for appropriate load redistribution and improved reinforcement (Muthe et al. 2022). A bespoke nozzle capable of extruding biocomposites in pellet form allows for affordable material formulation customization. Zhao et al. have developed large-scale AM (high deposition rate and build volume) with six degrees of freedom of movement for biocomposite materials. This allows for the faster and less expensive printing of complicated structures. These qualities might open up new possibilities for producing high-performance biocomposites. However, the technique is not cost-effective (Zhao et al. 2019b). The invention of methods for making 4D printed lightweight biocomposites can bring a novel approach to include self-sensing and actuation (autonomous and shape-morphing) properties in materials using locally available resources. This feature reduces the number of structural components, assembly time, material, and energy requirements in production, which are typical of electro-mechanical devices. The applications for these structures include construction such as solar tracking and shading, aircraft, automobiles, healthcare like architectural skin systems, and marine engineering (Correa et al. 2020; Le Duigou et al. 2017). Biofibers with hygroscopic qualities such as coir, jute, flax, or kenaf cause swelling that makes them operate as actuators. Therefore, it is necessary to create hygro-morph biocomposites with the ideal fiber volume content, especially when using a polymer that is hypersensitive to moisture. Better extrusion without blockage is possible with an ideal design.

## Conclusions

The use of 3D printing technology in the development of biological composites has resulted in major developments in sustainable production. The reuse of biomass for 3D printing reduces the energy content, which results in significant energy savings and economic gains. Despite the challenges, recycling in 3D printing benefits the environment by lowering waste and resource consumption. Biomass recycling in 3D printing can result in substantial energy consumption, greenhouse gas emissions, and production of waste when compared to traditional manufacturing processes and virgin resources. The utilization of renewable and waste resources like biopolymers and biomass promotes environmental sustainability. The analysis highlighted the potential of 3D-printed biocomposites to reduce their environmental footprint while also encouraging a circular economy. It enables the development of sustainable composites and the utilization of trash as resources in replacement for virgin materials. Recycling has an extensive impact on 3D-printed objects' mechanical properties, molecular structure, and chemical composition. While specific characteristics may have compromises and limitations, recycling paves the way for more sustainable production techniques and the development of novel materials with enhanced functionality. The reinforcement of biomass has shown significant improvements in mechanical properties. However, when the fractions of reinforcements increase, the strength of the composites decreases due to increasing brittleness, crack development, and agglomeration. As a result, research was needed to determine the best way to use biomass while maintaining the qualities of biocomposites.

The study on optimizing 3D printing parameters has discovered key factors that have a major effect on product quality and mechanical properties. Extrusion temperature, feed rate, nozzle diameter, raster angle, and printing speed influence the strength, ductility, crystallinity, and geometric correctness of printed products. The optimization of these elements leads to improved mechanical performance. One of the obstacles in using biomass is filament deterioration, which causes printing troubles and has an impact on its mechanical attributes. The inclusion of additives in polymer recycling and 3D printing has demonstrated significant promise for enhancing mechanical properties, thermal stability, adhesion, and dimensional stability. This review provides a thorough overview of biomaterials, processing procedures, characteristics, characterization methods, and future prospects for biocomposites. The purpose was to provide a thorough grasp of biocomposites' preparation, processing, and characterization. Lightweight, energy-efficient, health advantages, being biodegradable, sustainability, plentiful availability, and low cost render biocomposites as the successful candidate for a variety of applications and for protecting the bionetwork from the harmful effects of synthetic materials. Interfacial adhesion, stacking sequence, processing conditions, and other factors all have an impact on biocomposites' mechanical properties. Fiber dispersion and interfacial bonding can be improved by studying their basic morphology. The use of less common biomaterials can create a new value chain for biomass. Biocomposite materials can bring immediate advantages to rural communities, especially in underdeveloped areas where these resources are plentiful. The variability and hydrophilicity of biocomposites create additional challenges in the manufacturing and widespread utilization. The main challenges include variations in fiber quality, poor interfacial adhesion between fiber and matrix, poor fiber dispersion, water absorption, and so on. The possible fields of research for matrix materials include the manufacture and fabrication of novel cost-effective biopolymers with superior mechanical properties, transportation stability, storage and service life, lower processing temperatures, thermal stability, and recyclability. Some fewer prevalent biopolymers exhibit better tensile moduli than artificial polymers; thereby, they can be utilized to bridge the gap among artificial and traditional biodegradable polymers, which have restricted the mechanical capabilities and are costly. Novel uses emerge when biomaterial-based products become more dimensionally stable, moisture-resistant, durable, and fireproof.

Biocomposites have excellent specific mechanical properties, although there is considerable variety. This disadvantage can be overcome by utilizing modern manufacturing techniques. AM using fiber-reinforced polymer materials has a distinct set of prospects for advancement, particularly in the use of eco-friendly and multifunctional biobased materials for automotive, aerospace, pharmaceutical, and packaging industries. Although AM is a low-cost, scalable, and environmentally friendly process, it lacks rigorous procedures and standards restricting its ability to make high-quality objects. Another issue for AM system regulators and developers is the creation of safe software, considering that the vast majority of currently available systems are open source. Other concerns in AM include developing a more sustainable supply chain and improving waste management techniques. Commercialization of these materials is expected to grow in the future as people become more environmentally concerned, with the advancement of effective manufacturing methods and new applications. The primary impediment to biomaterials commercialization is a lack of significant research activity in developing countries, whereas these materials are plentiful. Despite the renewability and recyclability of biocomposites, researchers must address issues concerning norms and product standards for these materials. Biocomposites designed for structural purposes must comply with regulations for large-scale waste treatment.

## Future outlooks

Although biocomposite materials are gaining acceptance throughout scientific and industrial communities, the challenge remains in effectively replacing synthetic composites with biocomposites that have equivalent structural and functional characteristics. Because of their inherent low mechanical and thermal properties, biocomposites may prove challenging to fully replace typical artificial composites. The major actions for accomplishing the goal of high-performance biocomposites will include raw material verification, extraction procedure, sustainable crop development, biocomposites interfacial characteristics execution, material preparation and product manufacturing, secure service life, and creation of products. However, few studies have been conducted on multi-fiber reinforcements and polymer blends, although the fact that these materials can provide greater flexibility in terms of construction and adaptation. This topic warrants additional exploration due to the potential for novel uses. Additionally, more research is needed into product development and performance evaluation, as well as the effect of environmental aging on the failure mechanics caused by thermomechanical–chemical processes in biocomposites. Biocomposite materials must have high performance, durability, dependability, and serviceability in order to be extensively adopted.

The research on the 3D-printed biocomposite is potent for the transformation of industries from an open loop system to a closed loop system. Future research must concentrate on the modification of the fillers and fibers’ characteristics for better interactions with the matrices. The optimization of the 3D printing parameters can improve the properties of the 3D-printed biocomposites without compromising biodegradability. The integration of the nano-sized reinforcement can open avenues for sustainable alternatives to conventional composites. The digital design and simulation of the composite models through the implementation of artificial intelligence and machine learning must anticipate the process-property behavior. There is an urge for the systematic assessment of repeatability, cost-effectiveness, and compatibility with the conventional 3D printer. The fabrication of biobased filament via the recycling of plastics and agricultural waste can promote sustainability. The integration of LCA and end-of-life assessment must be performed to ensure the advantageous nature of the environment. The standardization of the protocols, strategies, and regulatory frameworks must be implemented to increase the application of 3D-printed biocomposites. The convergence of AM techniques, material science, and sustainable engineering can establish 3D-printed composites as advanced functional materials. The recent studies have supported the advancement of 3D-printed biocomposites. The natural fiber-based composites have shown the PLA-based flax, hemp, and jute fibers to increase the mechanical properties of the conventional petroleum composites. An improvement in the tensile strength with the incorporation of 10 wt. % of rice husk has been exhibited when compared to the neat PLA. The LCA of biocomposite filaments derived from agricultural residue has demonstrated a reduction in the carbon footprints by 30–50% in comparison to the conventional ABS or nylon filament. A balance in printability and eco-efficiency at higher loading can lower the environmental impacts, which decreases the dimensional accuracy. The lignin or cellulose-based composites have demonstrated the viability of the 3D-printed composite for automobile interiors and furniture where the considerations for recyclability and lightweight are significant. The optimizations in the printing parameters and filler content can influence the porosity and layer adhesion, expanding their functional use. The PLA wood flour composite has demonstrated an approximately 70% reduction in the embodied energy.

More research is needed to fully understand the potential of 3D printing in employing biodegradable plastic and biomass for circular economy applications. A study on the use of innovative sources of biocarbon reinforcement and treatment processes to increase the quality and compatibility of biocarbon for 3D printing can be conducted. Furthermore, more research is needed to discover how interlayer bond strength and printing errors affect the mechanical properties of 3D-printed products. As the use of recycled materials in 3D printing increases, it is vital to examine the entire life cycle of these products. The integration of artificial intelligence (AI) and machine learning (ML) techniques into 3D printing and recycling has the potential to significantly improve process control, material generation, reinforcements, and part quality. The combination of AI and machine learning can increase the precision and efficiency of recycling and 3D printing operations, ultimately boosting process control, material creation, filament, and part quality.

## Data Availability

Data sharing does not apply to this article as no new data were created or analyzed in this study.
